# FGF8 induces bone and joint regeneration at digit amputation wounds in neonate mice

**DOI:** 10.1016/j.bone.2025.117663

**Published:** 2025-10-03

**Authors:** Ling Yu, Mingquan Yan, Sarah M. Wolff, Joseph D. Knue, Hannah M. Smith, Connor P. Dolan, Ken Muneoka, Selim Romero, James J. Cai, Carissa Yun, Devon J. Boland, Regina Brunauer, Lindsay A. Dawson

**Affiliations:** a Department of Veterinary Physiology and Pharmacology, College of Veterinary Medicine and Biomedical Sciences, Texas A&M University, College Station, TX, 77843, USA; b Department of Veterinary Integrative Biosciences, College of Veterinary Medicine and Biomedical Sciences, Texas A&M University, College Station, TX, 77843, USA; c Texas A&M Institute for Genome Sciences & Society, College Station, TX, 77843, USA; d LBG Ludwig Boltzmann Institute for Traumatology, The Research Center in Cooperation with AUVA, 1200, Vienna, Austria; e Austrian Cluster for Tissue Regeneration, 1200, Vienna, Austria

**Keywords:** FGF8, Endochondral ossification, Joint regeneration, Joint morphogenesis, Cartilage, Bone

## Abstract

Due to increases in vascular diseases, the incidence of limb loss is predicted to more than double in the next quarter century. Therefore, developing a greater understanding of the latent regenerative capacity in mammals is a significant and growing goal. Mammals, including humans and mice, have limited regenerative capacity following limb amputation, with regenerative responses restricted to amputations transecting the distal digit tip (P3). Unlike P3, amputations of the adjacent skeletal segment, the middle phalanx, P2, are non-regenerative and result in bone truncation and soft tissue scar formation. As such, P2 amputation is a simple yet powerful model to test strategies for inducing mammalian musculoskeletal regeneration from an otherwise non-regenerative amputation plane. Here, we report that Fibroblast Growth Factor 8 (FGF8) drives synovial joint regeneration at P2 amputation wounds in neonate mice. This response is characterized by the regeneration of a synovial cavity, a skeletal nodule lined with articular cartilage, and tendon and ligament regeneration. FGF8 also induces cartilage formation on the P2 stump that serves as a template for partial P2 bone regeneration, thus FGF8 drives the composite regeneration of stump and joint tissues. FGF8-induced joint regeneration is associated with the upregulation of several, but not all, genes that characterize joint development, and is morphologically distinct from digit joint development. Lineage tracing studies demonstrate that cells at the amputation wound contribute to the regenerated joint structures. These studies provide evidence that the otherwise non-regenerative P2 amputation wound possesses tremendous regenerative capacity that is dormant under normal circumstances.

## 1. Introduction

It is estimated that 1 in 190 people in the United States live with limb loss, and due to increases in vascular disease, the incidence of limb loss is predicted to more than double by the year 2050 [1]. Limb amputations are associated with increased depression [2], decreased quality of life [3], decreased mobility [4], and an increased economic burden [5]. As such, developing a greater understanding of the latent regenerative capacity in mammals is a significant goal. Importantly, endogenous extremity regeneration responses are found in mammals, as amputations of the distal region of the fingertip can regenerate in humans [6–8], as can the distal digit tip (the terminal phalanx, P3) in mice [8–12]. P3 regeneration is characterized by complete bone restoration and is mediated by a structure called the blastema [10]. The blastema is a transient population of proliferating stem/progenitor cells that differentiate to re-form the tissues lost by amputation [10,13,14]. Unlike P3, amputations of the adjacent skeletal element, the middle phalanx, P2, are non-regenerative and result in bone truncation and soft tissue scar formation [15–17].

The amputation-level-dependent regenerative capacity of the digit offers it tremendous utility as a model system for devising strategies to induce mammalian tissue regeneration, in that knowledge gained from investigating P3 regeneration drives the selection of regenerative agents to screen at the P2 amputation wound. For example, given that Bone Morphogenetic Proteins (BMPs) and BMP receptors are expressed in the P3 blastema and are required for P3 bone regeneration [18], we demonstrated that BMP2 induces patterned P2 bone regeneration in neonate [16,19] and adult [20] mice, that can be further scaled up to limb amputations [16,21]. To facilitate bone regeneration at amputation wounds, BMP2 induces the formation of a transient cartilaginous structure, the endochondral ossification center (EOC) [16,20,22]. The EOC is comprised of chondrocytes at the stump apex that undergo hypertrophy in a polarized manner; hypertrophic chondrocytes are associated with the stump-EOC interface whereas chondrocyte proliferation is localized distally. The hypertrophic chondrocytes serve as a template for bone regeneration that extends directly from the amputation plane, and results in the complete restoration of amputated P2 length [16,20]. Of note, BMP2 regenerates only the bone that is amputated and does not regenerate other skeletal or joint structures. Accordingly, we next demonstrated that a different BMP, BMP9, induces synovial joint regeneration at P2 amputation wounds [22]. BMP9-induced joint regeneration involves the regeneration of a synovial cavity, a skeletal nodule lined with articular cartilage, and tendon and ligament regeneration, but does not involve regeneration of the P2 stump [22]. Indeed, we demonstrated that the sequential treatment strategy of first stimulating EOC formation with BMP2 followed by inducing joint regeneration via BMP9 drove the composite regeneration of both P2 stump and joint tissues [22]. Taken together, these findings demonstrate that the otherwise non-regenerative P2 amputation wound possesses tremendous regenerative capacity that is inactive under normal circumstances.

In addition to BMPs, Fibroblast Growth Factors (FGFs) play integral roles in musculoskeletal development and regeneration. Following limb amputation in highly regenerative animals, such as urodele amphibians, BMP7 and FGF8 secreted by the severed nerve functions to stimulate limb regeneration [23]. Using an induced accessory limb regeneration model in urodeles, the combined treatment of BMPs and FGFs (e.g., BMP2 plus FGF2 and/or FGF8) drive blastema and accessory limb formation after simple skin wounding [24,25]. A similar combination of BMPs and FGFs have also been shown to drive the regeneration of an accessory gill [26] and tail [27] in urodele amphibians. FGF signaling is also involved in P3 regeneration in mice and thus serves as a target growth factor family for potentially stimulating P2 regeneration. Indeed, FGF2 signaling has been shown to enhance blastema and osteoprogenitor proliferation during P3 regeneration [28], and has been recently discovered to induce the formation of a blastema-like structure at neonate P2 amputation wounds that can be further differentiated with sequential BMP2 treatment [29]. FGF2 treatment alone drives the formation of a joint-like structure at neonate P2 amputation wounds in approximately 30 % of digits [29]. Other FGFs drive regenerative outcomes at P2 amputation wounds, in that FGF8-treated iPSCs grafted into the adult P2 stump resulted in P2 skeletal elongation [30]. Given that FGF8 stimulated P2 bone elongation in adult mice [30], we narrowed our focus to screening FGF8 for regenerative outcomes at the neonate P2 amputation plane. In this study, we hypothesized that exogenous FGF8 could induce a multi-tissue regeneration response at neonate mouse P2 amputation wounds. To test this, we administered FGF8 at the P2 amputation wound and discovered that FGF8 drives the regeneration of a synovial joint-like structure. This response is characterized by the regeneration of a synovial cavity, a skeletal nodule lined with articular-like cartilage, and tendon and ligament regeneration. Intriguingly, FGF8 also induces EOC formation on the P2 stump that serves as a template for partial P2 bone regeneration. Therefore, FGF8 drives the composite regeneration of stump and joint tissues. We provide evidence that FGF8-induced joint regeneration is associated with the upregulation of some, but not all, genes that characterize joint development, and that joint regeneration is morphologically distinct from digit joint development. Lastly, lineage tracing studies demonstrate that cells at the amputation wound contribute to the regenerated joint structures. These studies provide evidence that regenerative failure after amputation is not due to a lack of cells that can participate in regenerative responses. Instead, we propose that regenerative failure is a consequence of absent and/or attenuated pro-regenerative signaling at the amputation site.

## 2. Methods

### 2.1. Mice, P2 amputation, and FGF8 treatment

Pregnant ICR mice (Charles River Strain Code: 022) were purchased from the in house breeding colony at the Texas A&M Institute for Genomic Medicine (TIGM). *Prg4*^*GFPCreERt2*^ mice [31] (JAX Stock # 022757) and *R26-tdTomato* mice [32] (JAX Stock # 007909) were purchased from the Jackson Laboratory and bred in house at TIGM. P2 digit amputations were carried out on hindlimb digits 2 and 4 at postnatal day 3 (PN3) [16,22]. P2 digit amputation transections the midportion of the P2 bone and entirely removes the P2/P3 joint, the sesamoid bone, the P3 bone and associated nail organ. Amputated digits were allowed to heal naturally, with would closure occurring on or prior to PN7. Following wound closure, implantation of a single agarose microcarrier bead (Affi-Gel Blue Gel beads, Bio-Rad, Hercules, CA) coated in FGF8 (500 ng/μl) or BSA (0.1 % in phosphate buffered saline) was implanted at PN7 into digits 2 and 4 using a sharpened tungsten needle [18], with each mouse yielding an n of up to 4 digits. To assess the distribution of FGF responsive cells soon after treatment, FGF8 or BSA treated samples were harvested 24 h after treatment and immunostained for phosphorylated-FGFR1 (p-FGFR1). Immunostaining revealed broad p-FGFR1 after FGF8 or BSA treatment, suggestive of endogenous FGF signaling within the digit. Importantly though, in FGF8-treated samples, p-FGFR1 was localized to the cells surrounding the induced cavity and the induced nodule, structures that are not present after BSA treatment (Supplementary Fig. 1). Amputations were performed on male and female mice in each litter, and all mice were treated similarly regardless of genotype. A power analysis determined a sample size of six digits was needed to detect a 17 % difference in cell number or bone length [33]. FGF8 treatment yields a ~ 61.5 % ratio of joint formation, therefore samples sizes consisted of *n* = 13–20 digits (4–5 mice) per experiment to yield an appropriate ratio, with sample sizes noted in the methods and results sections and in figure legends. Neonate mice were anesthetized by thermal cooling and adult mice were anesthetized by isoflurane inhalation (1.5–2.0 % in oxygen). Animals in each litter were randomly separated between FGF8 and BSA treatment groups and identified by ear notch. Male and female FGF8 or BSA treated digits were combined for analysis. Each experiment utilized at least two separate litters. At PN8, *Prg4*^*GFPCreERt2*+*/*−^*;R26-tdTomato*^+*/*−^ mice were injected (IP) with Tamoxifen (Sigma 5648; 10 mg/ml in corn oil, 10 μl per gram body weight; *n* = 20 digits (5 mice) per group). A subset of *Prg4*^*GFPCreERt2*+*/*−^*;R26-tdTomato*^+*/*−^ mice treated with FGF8 were injected with corn oil (n = 20 digits (5 mice)) as a control. Animal euthanasia was carried via carbon dioxide inhalation and verified via cervical dislocation. All animal use and techniques were approved on animal protocols *Wound Repair and Regeneration* - 2020–0267 (approval date 12/3/2020, expiration 10/31/23) and *Wound Repair and Regeneration* 2023–0204 (approval date 10/31/23, expiration 10/31/26) and received ethical approval from the Institutional Animal Care and Use Committee (IACUC) at Texas A&M University. These studies have been reported in line with the ARRIVE guidelines 2.0.

### 2.2. Tissue processing

For histology and immunohistochemistry, digits (*n* = 13–20 digits (*n* = 4–5 mice) per group per time point) were fixed in 10 % Neutral Buffered Formalin for 24–96 h at room temperature. For section in situ hybridization, digits were fixed in 4 % paraformaldehyde at 4 °C overnight. Digits were decalcified using Decalcifier I (Surgipath, Leica Biosystems, Richmond, IL) for 2 h (for digits collected prior to PN14) or 20 to 24 h (for digits collected after PN14) at room temperature. Digits were processed for paraffin histology, embedded in paraffin, and sectioned at 4 μm as previously described [34]. For section in situ hybridization, the *Prg4* (600 bp) antisense riboprobe was generated using the Digoxigenin-UTP transcription labeling Kit (Roche, Indianapolis, IN) as previously described [8] and counterstained with Alizarin Red (Sigma-Aldrich Co., St Louis, MO). For digit immunostaining, antigen retrieval has been described in great detail [34,35]. Primary antibodies were incubated overnight at 4 °C. Primary antibodies used include: Proliferating Cell Nuclear Antigen: mouse anti-PCNA (Abcam, Cambridge, UK; ab29, 1:1000 dilution); Collagen II: mouse anti-ColII (Acris Antibodies, San Diego, CA; AF5710, 1:200 dilution); Prg4: rabbit anti-Prg4 (LSBio, LS-B8236; 1:200); Sox9: rabbit anti-Sox9 (Abcam, ab185966, 1:500 dilution); p-FGFR1: rabbit polyclonal anti-FGFR1 (Phospho Y654) (Abcam, ab59194, 1:200 dilution); Aggrecan: rabbit anti-Acan (EMD Millipore, Billerica, MA; AB1031, 1:300 dilution); tdTomato: Goat anti-RFP (Origene, AB1140–100; 1:1000 dilution) or Rabbit anti-RFP (Abcam, ab62341; 1:100 dilution). Following primary antibody incubation, slides were washed with Tris-buffered saline with Tween 20 (Sigma-Aldrich Co.) and incubated in secondary antibodies for 45 min at room temperature [34], and counterstained with DAPI. For general histology, Mallory Trichrome staining was performed. Slide imaging was carried out using: the Olympus BX60 microscope and DP72 camera using the DP2-BSW software (Olympus America Inc., Center Valley, PA), the Olympus BX61 fluorescence deconvolution microscope using Slidebook software (Intelligent Imaging Innovations Inc., Denver, CO), or the Olympus VS120 microscope, with images processed using Fiji [36] and the BIOP VSI Reader [37]. Quantification of proliferating cells was performed on a medial section from each digit at 21 DPI; PCNA^+^ cells were manually counted and normalized to total DAPI cells. To determine statistical changes in cell proliferation, a one-way ANOVA was performed using GraphPad PRISM, version 9.5.1 (2023) (Graphpad Software, La Jolla, CA).

### 2.3. qRT-PCR RNA analysis

Total RNA was extracted from FGF8 (*n* = 16 digits; 4 mice) or BSA (n = 16 digits; 4 mice) treated digits from two individual litters at 24 h and 3 days post treatment using the RNeasy Plus Micro Kit (Qiagen) following the manufacturer’s instructions. Digits were pooled based on treatment and time of extraction. RNA quantification and quality control was performed using Nanodrop rations of 260/280 and 260/310. Following extraction, qRT-PCR was performed in triplicate with the SuperScript III Platinum One-Step qRT-PCR Kit w/Rox using the Eppendorf Realplex machine. The following Applied Biosystem Taqman primer sets (Thermo Fisher) were used: Col2a1 (Mm01309565_m1); Prg4 (Mm01284582_m1); Rpl12 (M02601627-gl); BMP2 (Mm01340178_m1); Wnt9a (Mm00460520_m1); Has2 (Mm00515089_m1); Cux1 (Mm01195598_m1); CD44 (Mm01277161_m1); Wnt4 (Mm01194003_m1); Osr1 (Mm00726877_m1); Osr2 (Mm00475202_m1); and GDF5 (Mm00433564_m1). Gene expression levels were normalized to the housekeeping gene, ribosomal protein L12 (RPL12), levels. Gene expression levels were analyzed using GraphPad PRISM (GraphPad Software, La Jolla, CA), using an unpaired *t*-test.

### 2.4. Single-cell RNA sequencing

Single-cell RNA sequencing (scRNA-seq) was performed 24 h post FGF8 (*n* = 20 digits; 5 mice) or BSA (n = 20 digits; 5 mice) treatment. Cell collection was carried out by removing the surrounding epithelium from the amputated digits and dissecting the tissue distal to the P2 stump, followed by placing the tissue into ice-cold PBS. Enzymatic dissociation was perform using 2 U/ml Liberase Blendzyme (37 °C, 4 h; Sigma-Aldrich Co.), with manual pipet trituration every 2 h. Cells from all FGF8 or BSA treated digits were pooled, thus it is likely the scRNA-seq includes FGF8 non-regenerative digits. Cells were filtered to remove aggregates, washed and resuspended in 0.8 % BSA in PBS.

Single cells were captured in Gel Beads-in-Emulsion (GEMs) using the Chromium Controller with Chromium Next GEM Single Cell 3’ Reagent Kits v3.1 (10× Genomics, Pleasanton, CA). Barcoded cDNA was generated through reverse transcription, followed by amplification and fragmentation. Dual-indexed sequencing libraries were constructed according to the 10× Genomics Single Cell 3′ v3.1 protocol. Libraries were quantified, quality-checked, and sequenced on an Illumina NextSeq 2000 (Illumina, San Diego, CA). FASTQ formatted sequencing reads were processed using CellRanger (v9.0.0) (10× Genomics, Pleasanton, CA) with default parameters and the GRCm39–2024-A transcriptome reference for UMI and gene expression quantification. All downstream analyses were performed in R.

Ambient and doublet captures were removed from the count matrices using Seurat (v5) [38] by applying filters: 200 < nFeature <7500 and mitochondrial gene count <15 %. Datasets from BSA and FGF8 treated samples were merged, and gene counts were normalized using the SCTransform algorithm [39]. The datasets were then integrated using the Canonical Correlation Analysis (CCA) method. Clustering was performed at a resolution of 0.05, identifying nine distinct clusters. Uniform Manifold Approximation and Projection (UMAP) was used for dimensionality reduction, and the resulting embeddings were visualized in 2D space. Cluster-specific and shared markers were identified using the FindConservedMarkers function in Seurat. Conserved markers were ranked by the difference in percentage of cells expressing each marker between clusters (pct.diff = pct.1 - pct.2), averaged across both samples. The resulting unique, conserved, and highly expressed markers were cross-referenced against the PanglaoDB [40] to assign cell types. To identify fibroblast sub-populations, the integrated Seurat object was subset to include only cells identified as fibroblasts. This subset was re-clustered at a resolution of 0.2, revealing six sub-populations.

The Seurat FindMarkers function was used to identify differentially expressed genes (DEGs). DEGs were defined as genes having a *p* (Benjamini-Hochberg corrected *p*-values) < 0.05 and |log_2_FoldChange| > 1. Pathway enrichment was conducted using both the GO [41] and KEGG [42,43] enrichment databases with the clusterProfiler (v4) [44]. Volcano plots and heatmaps were rendered with the EnhancedVolcano (v1.24) [45] and and ComplexHeatmap (v2.22) [46] packages, respectively.

### 2.5. Microcomputed tomography (μCT) scanning

μCT scanning was performed on age matched FGF8 (*n* = 16 digits, 4 mice), BSA (*n* = 13 digits, 4 mice), or unamputated P2 digits (*n* = 8 digit, 2 mice) using the vivaCT 40 (SCANCO Medical, Wayne, PA) with the same parameters we have described previously in detail [34,35]. Adult mice were anesthetized using isoflurane gas (1.5 % in oxygen) during the scan, and scans were carried in under 10 min. Image generation and analysis of bone length was performed using the BoneJ Plugin for Fiji [34,47]. Due to the radiolucent nature of PN3 and PN7 stage digits, immediate post amputation or post treatment bone length assessments via μCT were not made; instead, bone length comparisons were made between adult stage matched FGF8 vs BSA treated digits, and only using FGF8-treated samples that showed the presence of a skeletal nodule (*n* = 10/16 digits; 4 mice). As such, small differences in the P2 amputation plane could contribute to the trends observed. To determine statistical changes in bone length, a one-way ANOVA was performed using GraphPad PRISM.

## 3. Results

### 3.1. FGF8 treatment induces composite tissue regeneration at P2 amputation wounds

**Fig. 1.**
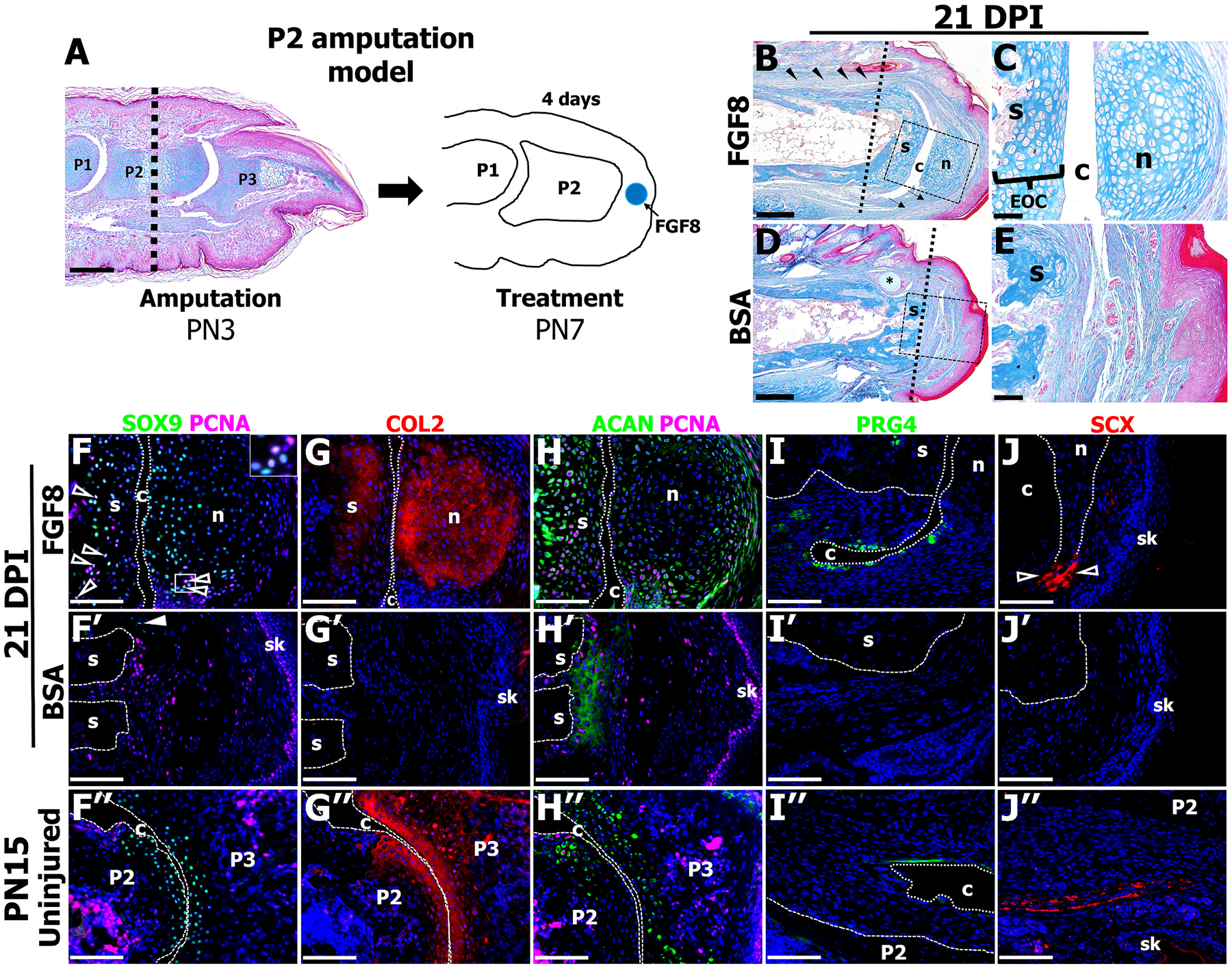
FGF8 induces composite tissue regeneration at neonate P2 amputation wounds. **A)** Mallory trichrome staining of an unamputated digit at postnatal day 3; dashed line indicates P2 amputation plane. Illustration of the P2 amputation wound at postnatal day 7 showcasing the blue bead coated in FGF8 or BSA as a control. **B)** Histological staining of a representative FGF8-treated digit at 21 days post implant (DPI) (*n* = 8/13 mice; 4 mice). Dashed line represents the original amputation plane, arrowheads indicate the regenerated dorsal ligament, and the arrows indicate the regenerated ligament attaching to the regenerated nodule (n). **C)** Inset of **B** (dashed box) illustrating the regenerated endochondral ossification center (EOC) on the P2 stump (s), the regenerated cavity (c), and the regenerated nodule. **D, E)** Histological staining of a representative BSA-treated digit at 21 DPI (*n* = 16 digits; 4 mice). Dashed line represents the original amputation plane, and the inset **E)** demonstrates fibrotic tissue capping the P2 bone stump. **F-J)** Immunohistochemical staining of FGF8-treated digits at 21 DPI. **F)** SOX9 and PCNA co-staining shows proliferating chondrocytes (open arrowheads and inset) within the EOC and the nodule. **G)** COL2 is localized to the stump and the nodule. **H)** Both the EOC and the nodule show broad ACAN immunostaining. **I)** PRG4 immunopositive cells line the regenerated cavity. **J)** SCX immunostaining identifies regenerated tendon cells (open arrowheads) abutting the regenerated nodule. **F′-J’)** Immunohistochemical staining of BSA-treated control digits at 21 DPI. **F′)** SOX9 and PCNA illustrates few chondrocytes (arrow) are present in the control digit. **G’)** COL2 is absent in the control digit. **H′)** ACAN and PCNA immunostaining shows diffuse ACAN localized to the bone stump and proliferating cells scattered throughout the fibrotic tissue. **I′-J’)** PRG4 and SCX are absent in the fibrotic tissue of control digits. **F**″**-J”)** Immunohistochemical staining of uninjured P2/P3 joints collected at PN15. **F**″**)** SOX9 and PCNA co-staining shows chondrocytes localized to the articular cartilage whereas cell proliferation is associated with the marrow cavities. **G”)** COL2 immunostaining is localized to the cartilage abutting the joint cavity. **H**″**)** ACAN immunostaining shows punctate staining within the joint cartilage, whereas PCNA is localized to the marrow regions. **I**″**)** PRG4 immunopositive line the uninjured joint cavity. **J”)** SCX is localized to the dorsal tendon subjacent to the P2 bone. Samples counterstained with DAPI. Distal is to the right, dorsal is to the top. * = bead; sk = skin. Scale bars: A, B, D = 200 μm; C, *E*-J” = 50 μm.

Middle phalanx (P2) amputation results in regenerative failure characterized by bone truncation and soft tissue scar formation. P2 amputation has been previously described [16,19,20,22]. Briefly, in neonate mice, P2 amputation at postnatal day 3 (PN3) eliminates the nail organ, the P3 and sesamoid bones, and the P2/P3 joint, while also bisecting the ventral tendon and dorsal ligament, and removing approximately 30 % of the P2 bone (Fig. 1A, amputation shown as a dashed line). To induce regenerative outcomes at P2, our previous studies utilized microcarrier beads implanted distal to the bone stump for targeted growth factor delivery [16,22]. We have shown that microcarrier beads transiently release growth factor over the span of 72 h [22]. Here, we used the same strategy; at 4 days post amputation (DPA), corresponding to PN7, an FGF8-coated microcarrier bead (500 ng/μl) or BSA control bead (0.1 % in PBS) was implanted into the amputation wound (Fig. 1A, blue dot). Digits were collected at 21 days post FGF8 or BSA implant (DPI) and processed for histological and immunohistochemical analysis. Mallory trichrome staining revealed a robust regeneration response induced by FGF8 treatment, characterized by a joint-like structure consisting of cartilage nodule (n) and cavity (c) formation, as well as ventral tendon (arrows) and dorsal ligament (arrowheads) regeneration (Fig. 1B, C; *n* = 8/13 (4 mice), 61.5 % frequency, split evenly between males and females). Our previous studies have shown that BMP2 induces chondrogenesis on the stump apex, and the chondrocytes are organized into an Endochondral Ossification Center (EOC) that facilities restoration of the amputated P2 length [16,22]. Importantly, FGF8 also induced chondrogenesis at the P2 stump (s) apex distal to the amputation plane (Fig. 1B, C, amputation plane shown as a dashed line), thus inducing the formation of an EOC-like structure. Comparatively, BSA treatment did not yield a multi-tissue regeneration response, and instead showed bone truncation at the amputation level (dashed line) and scar tissue capping the P2 stump (Fig. 1D, E, n = 16 digits; 4 mice). The 38.5 % of FGF8 non-regenerative digits parallelled the BSA response, in that they showed bone truncation at the amputation plane and soft tissue scar formation (Supplementary Fig. 2 A). Of note, both BSA and non-regenerative FGF8-treated digits were histologically analogous to digits that received a simple P2 amputation followed by no treatment and endogenous wound healing, as previously described in detail [17].

To determine if FGF8 treatment induced a multi-tissue joint regeneration response, immunostaining using markers for cartilage, synovial cavity, and tendon was performed on FGF8-treated digits that formed a joint or BSA-treated digits harvested at 21 DPI and compared to the unamputated PN15 joint. Double immunostaining for the early cartilage marker SOX9 and Proliferating Cell Nuclear Antigen (PCNA) revealed SOX9^+^/PCNA^+^ cells within the FGF8-induced EOC and localized to the periphery of the nodule (Fig. 1F, open arrowheads and inset). SOX9 immunostaining was polarized within the nodule, with SOX9^+^ cells primarily localized adjacent to the cavity (Fig. 1F). In contrast, BSA-control digits revealed few SOX9^+^ chondrocytes (arrowhead), and cell proliferation was dispersed within the fibrotic cap (Fig. 1F’), similar to the dispersed PCNA^+^ cells in non-regenerative FGF8-treated digits (Supplementary Fig. 2B). In the uninjured joint, SOX9 was localized to the P2/P3 articular cartilage whereas cell proliferation was largely associated with the marrow regions (Fig. 1F”). The cartilage marker Collagen Type 2 (COL2) showed broad staining in the FGF8-induced nodule and the EOC similar to the broad staining in the uninjured P2/P3 joint, whereas BSA-control digits lacked COL2 staining at the distal stump (Fig. 1G–G”). The cartilage marker Aggrecan (ACAN) and PCNA revealed robust co-staining of the EOC, whereas the nodule showed punctate ACAN staining (Fig. 1H). In BSA-control digits, ACAN staining capped the distal stump, potentially suggestive of fibrocartilage formation, as fibrocartilage expresses ACAN [48]. In the uninjured joint, punctate ACAN immunostaining was broadly localized to the P2/P3 articular cartilage (Fig. 1H”). Immunostaining for Proteoglycan 4 (PRG4), the joint lubricant expressed by synovial fibroblasts [49], identified PRG4^+^ cells lining the FGF8-induced cavity as well as the uninjured digit P2/P3 joint cavity, whereas PRG4 was absent at the stump of BSA-control digits (Fig. 1I–I”; representative area of Fig. 1I shown in Supplementary Fig. 3). Lastly, the tendon marker, Scleraxis (Scx), showed Scx^+^ cells abutting the nodule in FGF8-treated digits (Fig. 1J), corresponding to the regenerated ventral tendon attachments (Fig. 1B; arrows). Scx^+^ cells were not present in the distal stump of digits treated with BSA (Fig. 1J’). Scx^+^ immunostaining was broadly localized to the ventral tendon subjacent to the P2 bone in the uninjured digit (Fig. 1J”).

**Fig. 2.**
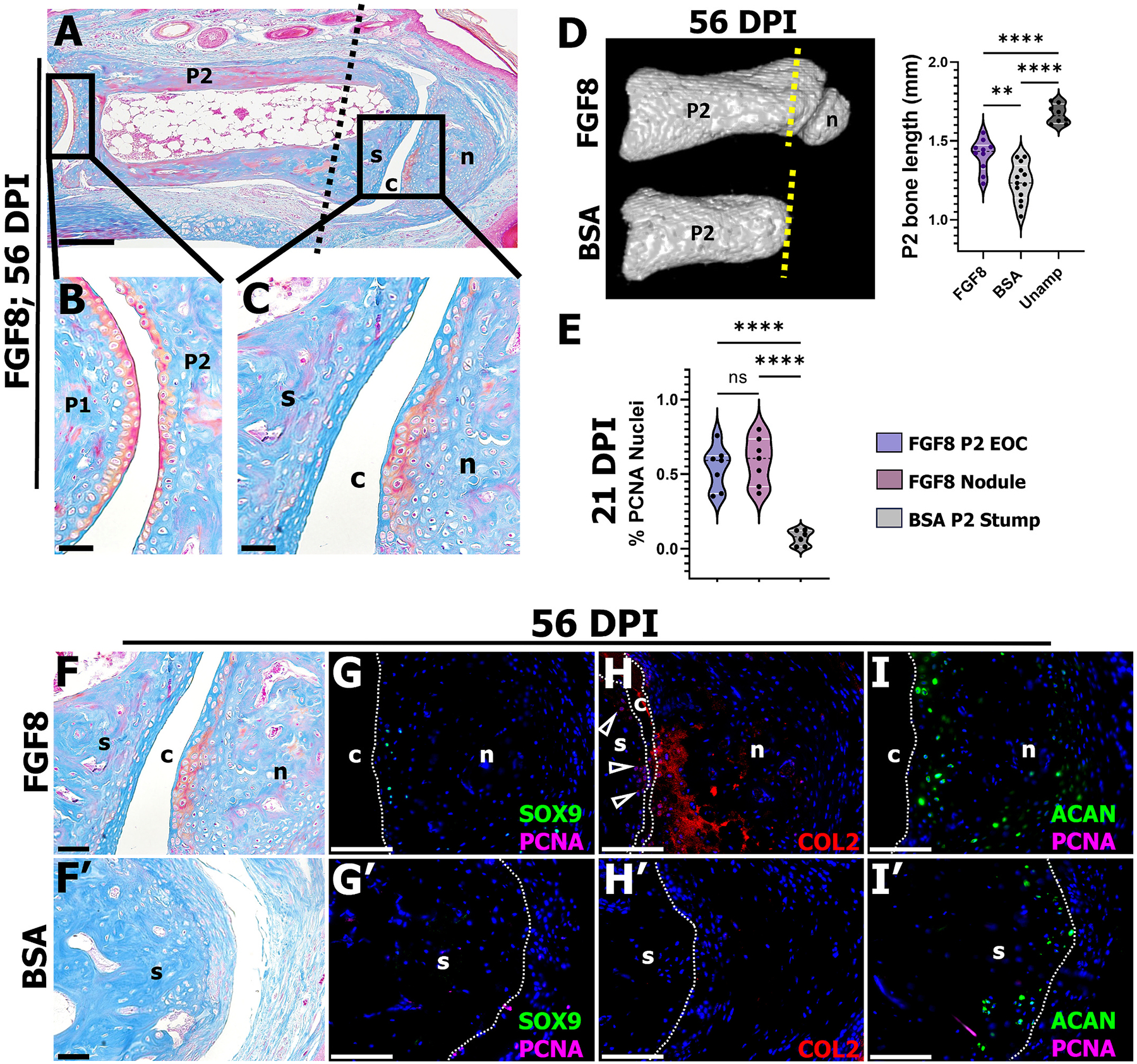
FGF8 induces partial P2 bone regeneration and stable joint regeneration. **A)** Representative Mallory trichrome stained FGF8-treated digit at 56 DPI comparing the uninjured P1/P2 joint **(B)** to the regenerated joint **(C)**. **D)** Representative micro CT renderings of FGF8 (*n* = 10; 4 mice) and BSA-treated digits at 56 DPI (*n* = 13; 4 mice); amputation plane shown as a yellow dashed line. Quantification of P2 bone length revealed FGF8 stimulated partial P2 stump regeneration (one-way ANOVA; **** *p* < 0.0001, ** *p* < 0.01). **E)** Quantification of proliferating cells revealed the FGF8-induced nodule and EOC (*n* = 7 digits; 4 mice) show enhanced proliferation compared to the BSA-treated (*n* = 6 digits; 3 mice) P2 stump at 21 DPI (one-way ANOVA; **** *p* < 0.0001). **F)** Representative Mallory trichrome stained FGF8-treated digit at 56 DPI. **G-I)** Immunohistochemical staining of serial sections of FGF8-treated 56 DPI sample shown in **F**. **G)** SOX9 and PCNA immunostaining revealed SOX9^+^ cells lining the cavity (c) and no cell proliferation. **H)** COL2 immunostaining localized to nodule (n) cells lining the cavity and few stump (s) cells lining the cavity (open arrowheads). **I)** ACAN^+^ cells localized to the periphery of the nodule. **F′)** Representative Mallory trichrome stained BSA-treated digit at 56 DPI. **G’-I′)** Immunohistochemical staining of serial sections of BSA-treated 56 DPI sample shown in **F′**. **G’)** SOX9^+^ cells are absent and there are few proliferating cells after BSA treatment. **H′)** BSA-treated digits lacked COL2 localization. **I′)** ACAN^+^ cells are present on the P2 bone stump at 56 DPI. Samples counterstained with DAPI. Distal is to the right, dorsal is to the top. Scale bars: A = 200 μm; B, C, E-H′ = 50 μm.

To gain insight into the maturation of the regenerated structures, FGF8 (*n* = 10/16 digits (4 mice), 62.5 % joint frequency) and BSA-control (*n* = 13; 4 mice) digits were harvested at 56 DPI. Histological and microcomputed tomography (μCT) analysis demonstrated that the nodule (n) ossified centrally (Fig. 2A, D), whereas the cartilage adjacent to the synovial cavity (c) matured into histologically distinguishable articular-like chondrocytes (Fig. 2A, C) akin to the uninjured P1/P2 joint articular cartilage (Fig. 2B). We have previously shown that BMP9 treatment induced the formation of a hemijoint, in that the nodule-associated cartilage differentiated into articular cartilage, whereas cells along the stump did not [22]. After FGF8 treatment, we also noted that the stump (s) cartilage did not show histologically distinguishable articular-like chondrocytes by 56 DPI (Fig. 2C). Given that the stump forms an EOC, and at 56 DPI the P2 stump has ossified, we questioned if the transient stump EOC-chondrocytes serve as a template for bone regeneration. Indeed, μCT scanning demonstrated that the FGF8-induced EOC stimulated 14 % restoration of the P2 bone length compared to BSA-controls (amputation plane shown as a dashed line in Fig. 2A, D; quantification performed on FGF8-treated digits showing the presence of a nodule, n = 10 of the 16 total treated digits; 4 mice), yet did not fully restore bone length to the unamputated level (Fig. 2D; one-way ANOVA; **** *p* < 0.0001, ** *p* < 0.01; *n* = 8 unamputated digits (2 mice)). We speculated that cell proliferation localized to the EOC, as shown in Fig. 1F and H, was associated with the increase in P2 bone length post FGF8 treatment. To investigate this, we quantified PCNA^+^ cells localized to both the FGF8-induced EOC and the FGF8-induced nodule (*n* = 7 digits; 4 mice) and compared that to cell proliferation localized to the distal P2 stump of BSA-control (*n* = 6; 3 mice) digits at 21 DPI. Cell proliferation was similar between the FGF8-induced EOC and the nodule, yet both showed enhanced proliferation compared to the P2 stump of BSA-treated control digits (Fig. 2E, one-way ANOVA; **** *p* < 0.0001). Like the 56 DPI histological staining, immunostaining for SOX9, COL2, and ACAN demonstrated that nodule-associated chondrocytes were primarily localized adjacent to the cavity (Fig. 2G–I), and that few COL2^+^ cells were present on the P2 stump (open arrowheads) after FGF8 treatment (Fig. 2H). BSA-control digits showed fibrous tissue capping the bone stump (note the space in the tissue is not a synovial cavity, but an artifact of tissue processing and sectioning) and no distinguishable chondrocytes (Fig. 2F’). BSA-control digits lacked SOX9^+^ and COL2^+^ cells yet showed robust ACAN immunostaining at the stump apex further suggestive of fibrocartilage formation [48] (Fig. 2G’–I′). Collectively, these data support the conclusion that FGF8 treatment induced a composite tissue regeneration response at the otherwise non-regenerative P2 amputation wound, characterized by the regeneration of a synovial joint with stable articular-like cartilage and partial stump bone restoration facilitated by EOC proliferation.

### 3.2. FGF8-induced joint regeneration taps into some, but not all, genes that characterize joint development and is morphologically distinct from digit joint development

**Fig. 3.**
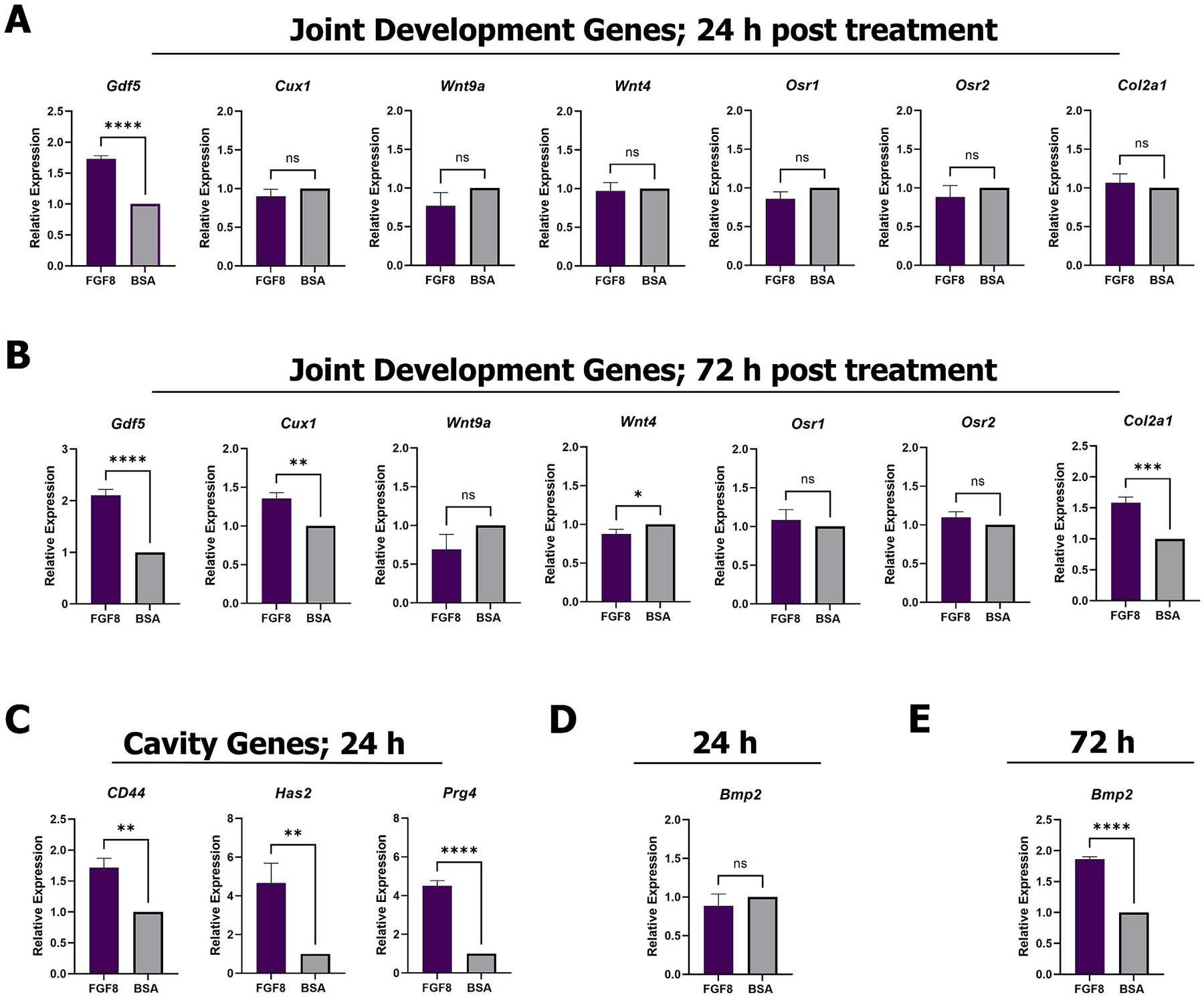
FGF8 modulates gene expression of joint development, cavity development, and *Bmp* genes in P2 amputation wounds. Gene expression analysis using qRT-PCR of **A, B)** joint development and interzone formation genes, and **C)** joint cavitation and lubrication genes. **D, E)** FGF8 treatment had no effect on *Bmp2* at 24 and enhanced *Bmp2* at 72 h. Gene expression was normalized to *Rpl12*. FGF8: n = 16 digits pooled (4 mice); BSA: n = 16 digits pooled (4 mice). Unpaired *t*-test; **** *p* < 0.0001, *** *p* < 0.005, ** *p* < 0.01, * *p* < 0.05; ^ns^*p* > 0.05.

The induction of a joint after amputation raises the intriguing possibility that FGF8-induced joint regeneration recapitulates the gene expression of joint development. During limb development, the structure that will eventually become the skeleton is initially comprised of a contiguous cartilaginous rod, the cartilaginous anlagen, and joint development proceeds within the cartilage anlagen via distinct regions called the joint interzone [50,51]. Incipient joint formation is marked by the expression of *Gdf5* [52], *Cux1* [53], *Wnt9a* (formerly *Wnt14*) [54], *Wnt4* [55], *Osr1* and *Osr2* [56,57], the downregulation of *Col2a1* [58], the upregulation of the cavity associated genes *Has2* [59] and *CD44* [60], as well as the upregulation of the joint lubrication gene *Prg4* [49]. We assayed the expression of these candidate genes at early time points post FGF8 (*n* = 16 digits pooled; 4 mice) or BSA (n = 16 digits pooled; 4 mice) treatment (Fig. 3). At 24 h, FGF8 treatment upregulated *Gdf5* expression, but did not alter *Cux1*, *Wnt9a*, *Wnt4*, *Osr1*, *Osr2*, or *Col2a1* expression (Fig. 3A; unpaired *t*-test; **** *p* < 0.0001, *** *p* < 0.005, ** *p* < 0.01, * *p* < 0.05; ^ns^*p* > 0.05; cycle threshold (Ct) values shown in Supplementary Fig. 4.). At 72 h, FGF8 upregulated *Gdf5*, *Cux1*, and *Col2a1* expression, had no effect on *Wnt9a*, *Osr1*, and *Osr2* expression, and downregulated *Wnt4* expression (Fig. 3B). At 24 h, expression of *CD44*, *Has2*, and *Prg4* was upregulated after FGF8 treatment (Fig. 3C), providing evidence that the onset of cavitation and cavity lubrication are an early event following FGF8 treatment. These data suggest that FGF8-induced joint regeneration may tap into gene expression of joint development, but that it is not simply a genetic replica of joint development in the context of a wound environment.

We have previously shown that sequential BMP2 and BMP9 treatment induced EOC and joint regeneration at the P2 amputation wound [22]. Due to the similarities in regenerative outcomes between sequential BMP2 and BMP9 treatment and FGF8 treatment, we next sought to determine if FGF8 upregulated *Bmp2* to induce the similar responses. At 24 h, *Bmp2* was unchanged (Fig. 3D), yet by 72 h, *Bmp2* expression was enhanced (Fig. 3E). These data point to the interesting possibility that FGF8 could stimulate EOC formation via upregulation of *Bmp2*.

**Fig. 4.**
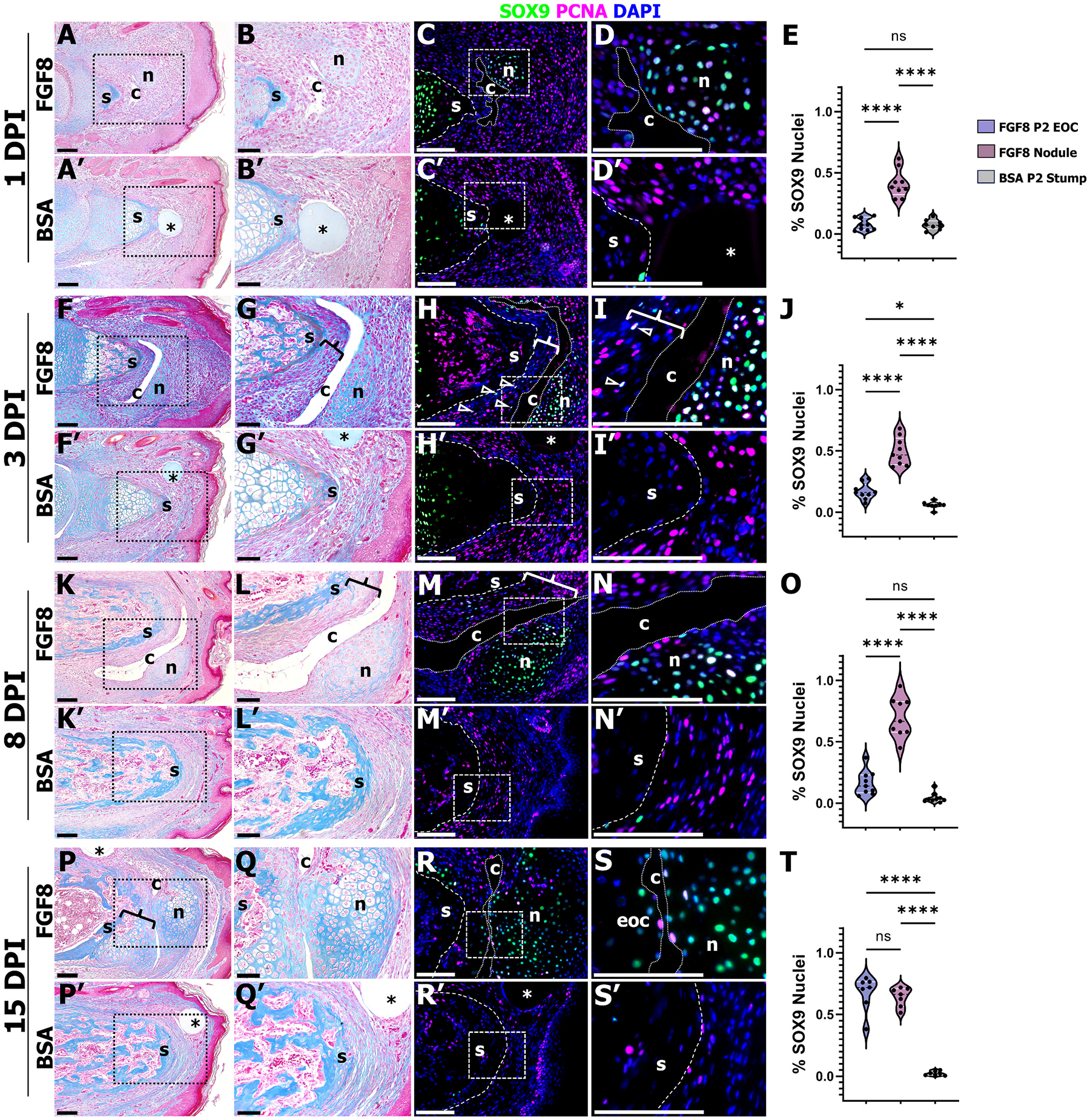
FGF8 stimulates nodule and cavity formation first, and secondarily stimulates EOC formation. Images on the left are representative samples of FGF8 and BSA treated digits stained with Mallory trichrome. Dashed boxes denote inset shown in the central column of images. Images on the right are serial sectioned samples immunostained with SOX9 and PCNA, and counterstained with DAPI. **A-E)** FGF8 stimulates disorganized cavity (c) and early SOX9^+^ nodule (n) formation at 1 DPI, whereas BSA demonstrates typical amputation wound healing leading to fibrosis. **F-J)** The FGF8-induced nodule and cavity continue to mature, while the EOC (bracket) shows few SOX9^+^ chondrocytes (open arrowheads) at 3 DPI. BSA-treated digits lack cavity, nodule and EOC formation. **K**–**O**) _At 8_ DPI, the chondrogenic nodule and cavity are prominent, whereas the EOC (bracket) is immature with few SOX9^+^ cells. BSA-treated digits illustrate continued fibrotic scar formation. **P-T’**) By 15 DPI, the FGF8-induced EOC (bracket) has differentiated into chondrocytes, and both the EOC and the nodule are comprised of SOX9^+^ cells. BSA treated digits show stump wound healing and fibrotic scar formation capping the stump. Histological analyses: FGF8: n = 16–20 digits per time point (4–5 mice per time point); BSA: n = 16 digits per time point (4 mice per time point). Immunostaining quantification: 1 DPI: FGF8 = 9 digits (4 mice); BSA = 8 digits (4 mice). 3 DPI: FGF8 = 9 digits (4 mice); BSA = 8 digits (4 mice). 8 DPI: FGF8 = 9 digits (5 mice); BSA = 9 digits (4 mice). 15 DPI: FGF8 = 7 digits (4 mice); BSA = 7 digits (4 mice). Distal is to the right, dorsal is to the top. * = bead. Scale bars: Left colum*n* = 200 μm; Middle and Right columns = 50 μm. One-way ANOVA; **** *p* < 0.0001, * *p* < 0.05; ^ns^*p* > 0.05.

**Fig. 5.**
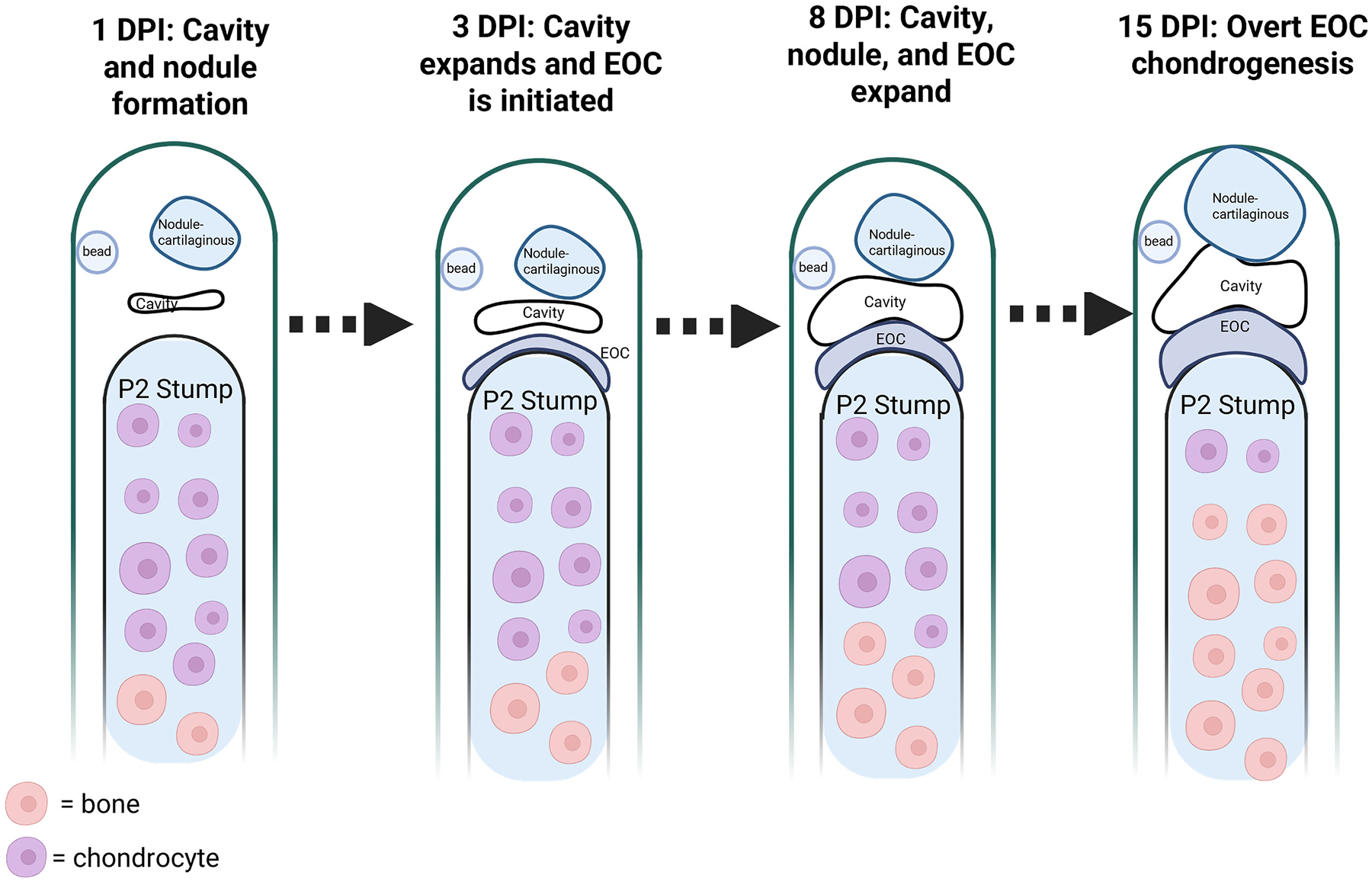
Illustration of FGF8-induced joint morphogenesis. Distal is to the top, proximal to the bottom. Image created in BioRender. Wolff, S. (2025) https://BioRender.com/t7q74fq.

Given that FGF8-induced joint regeneration was characterized by the upregulation of several genes associated with joint development, we next questioned to what extent the morphology of induced-joint regeneration was analogous to digit joint development. Digit joint development occurs within the interzone and essentially interrupts the cartilaginous anlagen. Developmental digit joint cavitation has been eloquently described; the nascent cavity initiates as a series of microcavities that form within the interzone, and merge over the course of several days to form the joint cavity proper [61]. To investigate the early stages of digit joint regeneration, FGF8 (*n* = 16–20 digits per group (4–5 mice) and screened for the presence of nodule) or BSA-treated digits (n = 16 digits per group (4 mice)) were harvested at 1, 3, 8, and 15 DPI (Fig. 4). FGF8-induced joints harvested at 1 DPI demonstrated that cavity formation initiated as a disorganized space adjacent to the early nodule (Fig. 4A, B), whereas nodule and cavity formation was absent after BSA treatment (Fig. 4A’, B′). Quantification of SOX9 immunostaining at 1 DPI showed that nodule chondrocyte differentiation occurred prior to stump EOC chondrocyte differentiation, as stump SOX9^+^ cells were restricted to the proximal P2 stump growth plate (Fig. 4C, D, E). Both FGF8 and BSA-treated digits showed broad cell proliferation throughout the wound site at 1 DPI (Fig. 4C, D’; Supplementary Fig. 5 A). By 3 DPI in FGF8-treated digits, the cavity had become organized into a single space localized distal to the early EOC (Fig. 4F, G), while control digits lacked cavity, nodule, and EOC formation (Fig. 4F’–I′). SOX9^+^/PCNA^+^ cells were localized to the nodule, whereas the EOC was several cell layers thick (bracket in Fig. 4F, G) but had few SOX9^+^ chondrocytes compared to the nodule (Fig. 4J), including several SOX9^+^/PCNA^+^ cells (Fig. 4H, I, arrowheads, Supplementary Fig. 5B). At 8 DPI, the nodule was largely comprised of SOX9^+^ cells and cell proliferation associated with the nodule was primarily localized to cells directly lining the cavity, whereas chondrocyte differentiation of the EOC (bracket) was again not robust at this stage (Fig. 4K–O). By 15 DPI, the EOC (bracket) had differentiated into chondrocytes (Fig. 4P, Q, R, S, T), and both the nodule core and the proximal EOC were undergoing chondrocyte hypertrophy. BSA-treated digits at both 8 and 15 DPI lacked nodule, cavity, and EOC formation, and instead were characterized by bone truncation and soft tissue scar formation that encased the stump (Fig. 4K’–N′, P′–S′). At all timepoints assayed, immunostaining quantification revealed no differences in cell proliferation, and no differences in the proportion of proliferating chondrocytes (Supplementary Fig. 5), thus FGF8 did not induce changes in chondrocyte proliferation, but rather induced chondrocyte differentiation in treated digits. These findings provide evidence that the FGF8-induced joint regeneration response is morphologically distinct from digit joint development, as digit joint development begins as an interruption of the contiguous cartilage rod, whereas the FGF8-induced joint is initially characterized by simultaneous cavity and nodule formation (and chondrocyte differentiation) early after treatment, and secondarily by EOC formation and chondrocyte differentiation, followed by expansion of the nodule, cavity, and the EOC (Fig. 5).

**Fig. 6.**
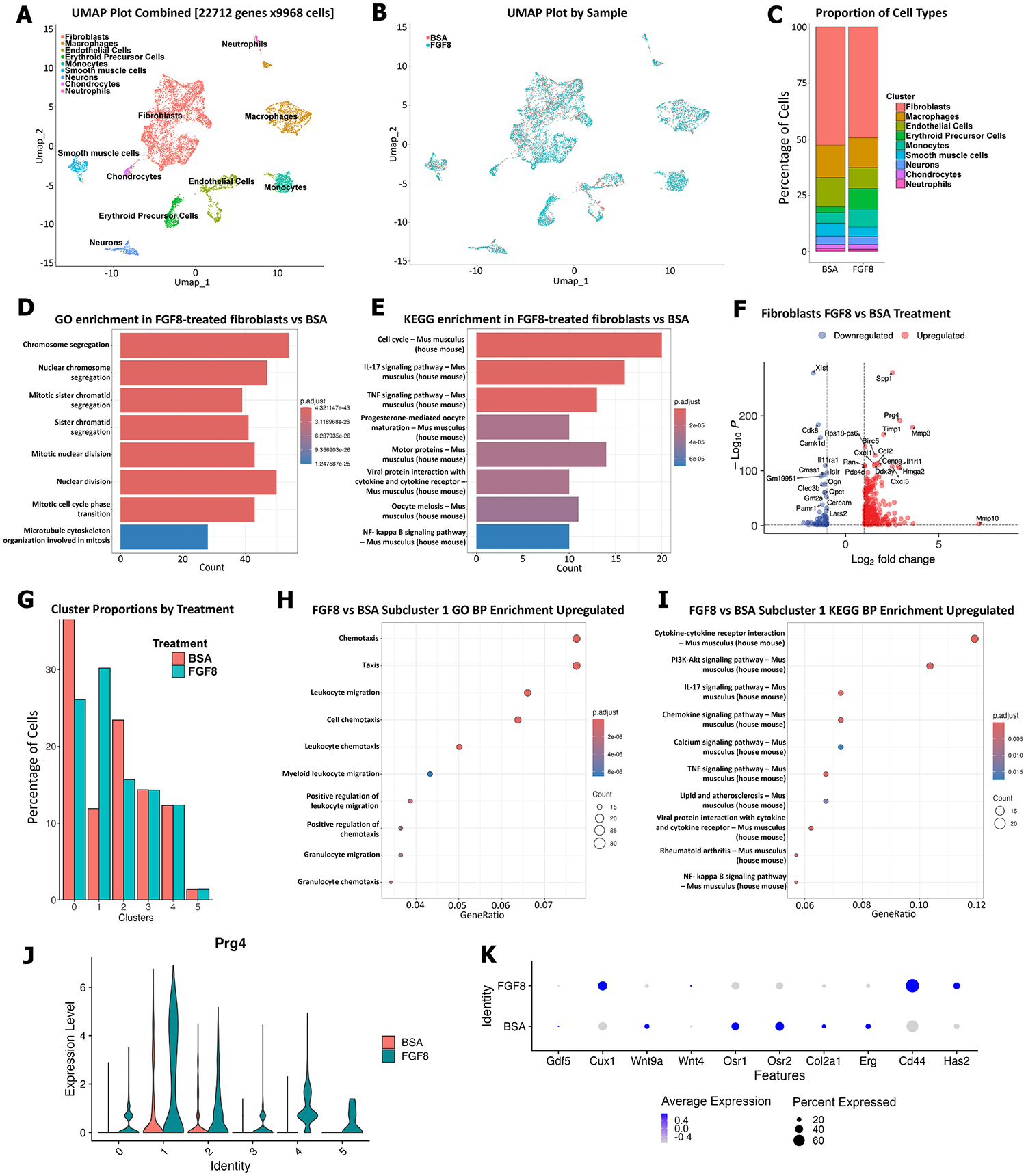
scRNA-seq identifies FGF8-driven enrichment of cell migration, proliferation, and ECM remodeling. **A, B)** UMAP plots of digit cells at 24 h post FGF8 (n = 20 digits pooled) or BSA (n = 20 digits pooled) treatment. **C)** Fibroblasts are the most abundant cell type. **D, E)** GO and KEGG enrichment specific to fibroblasts. **F)** Differential gene expression in FGF8 vs BSA treated fibroblasts. **G)** 6 overlapping fibroblast subclusters were identified between FGF8 and BSA datasets, with only subcluster 1 showing enhanced proportion of cells following FGF8 treatment. **H, I)** FGF8-upregulated GO and KEGG enrichment specific to subcluster 1. **J)** *Prg4* expression levels in the 6 fibroblast subclusters. **K)** Fibroblast joint and cavity marker expression, shown as dot plots.

Histological analysis indicated that cavity and nodule morphogenesis were an early event following FGF8 treatment, occurring within the first 24 h (Fig. 4A–C). To gain a deeper understanding of the transcriptional drivers of this response, we generated single-cell RNA sequencing (scRNA-seq) datasets at 24 h post FGF8 (*n* = 20 pooled digits; 5 mice) or BSA (n = 20 pooled digits; 5 mice) treatment. The FGF8 dataset yielded 6716 cells and the BSA control was comprised of 3252 cells. 9 overlapping cell clusters were identified (Fig. 6A, B), with fibroblasts the most abundant cell type (Fig. 6C). Given the abundance of fibroblasts, and consistent with studies identifying fibroblasts as a key component of P3 regeneration [62–64], we assessed differential gene expression across fibroblasts with an average Log2FC > 1 and identified 270 upregulated genes after FGF8 stimulation (Supplementary Table 1) and 84 downregulated genes (Log2FC < 1; Supplementary Table 2). Gene Ontology (GO) analysis identified enrichment of cell proliferation in the fibroblasts following FGF8 treatment (Fig. 6D). Similarly, KEGG enrichment analysis also identified cell proliferation, as well as general inflammatory pathways (Fig. 5E). Extracellular matrix (ECM) degradation and tissue histolysis are key components of diverse regenerative responses, including deer antler regeneration [65], P3 regeneration [10,35,66], and salamander limb regeneration [67–71]. FGF8 upregulated genes associated with tissue remodeling and ECM degradation and reorganization (*Spp1* [72], *Mmp3*, *Mmp10*, *Adam8* [73]), upregulated the MMP inhibitor *Timp1*, and downregulated *Adamts19*, a metalloproteinase linked to the suppression of cell migration [74] (Fig. 6F, Supplementary Tables 1 and 2). Next, we interrogated the fibroblasts to determine if distinct sub-populations were induced after FGF8 treatment. We subset and re-clustered the fibroblasts and instead we identified 6 fibroblast subclusters shared between FGF8 and BSA treatments (Fig. 6G; Supplementary Fig. 6A). Of the 6 clusters, 3, 4, and 5 showed a similar proportion of cells, whereas clusters 0, 1, and 2 showed differences in cell proportion with only cluster 1 enhanced after FGF8 treatment (Fig. 6G). Further analysis of fibroblast subcluster 1 identified enrichment of cell chemotaxis and migration (Fig. 6H), as well as enrichment of cytokine, chemokine, PI3K-Akt and inflammatory signaling, and intriguingly, enrichment of processes associated with rheumatoid arthritis (Fig. 5I). Collectively, these data suggest a complex regulation of cell proliferation, ECM remodeling, and cell migration during early FGF8-induced joint regeneration.

Like our qRT-PCR findings, *Prg4* was upregulated (Fig. 5F) and expressed in 60.9 % of FGF8-treated fibroblasts compared to 13.8 % of BSA-treated fibroblasts (Supplementary Table 1), as well as upregulated in all 6 fibroblast subclusters (Fig. 6J). We next probed the fibroblast population for the joint development markers that we had assessed by qRT-PCR analysis (Fig. 3). *Cux1* and *Wnt4* were enriched after FGF8 treatment, whereas *Gdf5*, *Wnt9a*, *Osr1*, *Osr2*, *Col2a1*, and *Erg* were enriched in BSA fibroblasts, again suggesting that FGF8-induced joint regeneration is not a replica of joint development in the context of a wound environment (Fig. 6K). Intriguingly, we had observed elevated *Gdf5* expression by qRT-PCR in FGF8 treated digits (Fig. 4) but not in FGF8-treated fibroblasts (Fig. 6K), therefore we assayed all cell populations for *Gdf5* and discovered that *Gdf5* was enriched in FGF8-treated chondrocytes compared to BSA-control samples (Supplementary Fig. 6B). Cavity formation genes (*Cd44* and *Has2*) were enriched after FGF8 treatment (Fig. 6K), in line with the histological evidence of cavity formation at 24 h post FGF8 treatment (Fig. 4A).

### 3.3. Cells at the amputation wound contribute to the regenerated joint structures

**Fig. 7.**
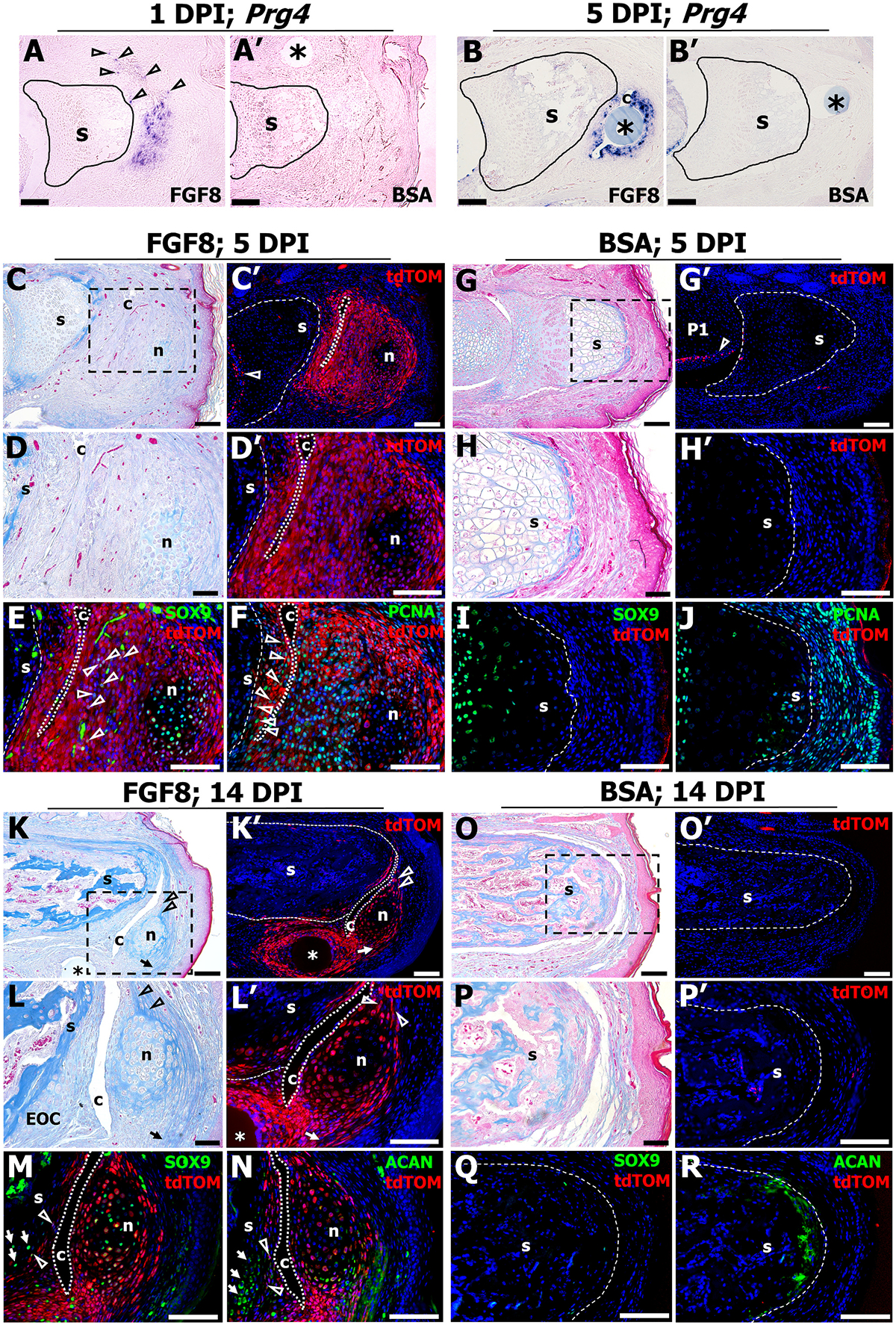
The FGF8-induced *Prg4*-lineage contributes to the regenerated structures. **A, A’)** Representative in situ hybridization samples illustrating *Prg4* transcripts at 1 DPI, and **B, B′)** at 5 DPI. **C)** Representative FGF8-treated sample at 5 DPI stained with Mallory trichrome. **C′)** Immunostaining for tdTOM demonstrating the *Prg4*-lineage contribution to the regenerating structures, as well as the endogenous *Prg4*-lineage localized to the P1/P2 joint (open arrowhead). **D**–**F)** Inset region of fig. **(C)** (dashed box). **D, D′)** Higher magnification images showing the newly formed cavity (c) and nodule (n) are derived from the *Prg4*-lineage. **E)** SOX9 and tdTOM immunostaining demonstrating chondrogenic differentiation of the nodule and sparse SOX9^+^ cells (open arrowheads) adjacent to the cavity. **F)** PCNA and tdTOM immunostaining illustrating robust cell proliferation (open arrowheads) in the early EOC associated with the stump (s) and polarized within the nodule. **G)** Representative BSA-treated sample at 5 DPI stained with Mallory trichrome. **G’)** Immunostaining for tdTOM demonstrating few immunopositive cells and endogenous *Prg4*-lineage localized to the P1/P2 joint (open arrowhead). **H-J)** inset region of fig. **(G)**, (dashed box). **H′)** TdTOM is nearly absent after BSA treatment. **I)** SOX9 is localized to the P2 growth plate, whereas few cells are immunopositive for tdTOM. **J)** PCNA immunostaining is robust within the P2 growth plate and the surrounding soft tissue. **K, K′)** Representative FGF8-treated sample at 14 DPI stained with Mallory trichrome **(K)** and for tdTOM **(K′)** showing the ligament attachments (open arrowheads) and the tendon attachments (arrow) to the nodule. **L, L’)** Higher magnification images showing the maturing regenerated structures are predominantly derived from the *Prg4*-lineage. **M)** SOX9^+^ cells are predominantly localized to the Prg4-derived nodule. **N)** ACAN and tdTOM immunostaining demonstrating some chondrocytes of the EOC (arrows) are not derived from the Prg4-lineage. **O, O′)** Representative BSA-treated sample at 14 DPI stained with Mallory trichrome **(O)** and for tdTOM **(O′)** showing regenerative failure and few tdTOM^+^ cells. **P, P′)** Higher magnification images showing regenerative failure and few tdTOM^+^ cells. **Q)** Few SOX9^+^ or tdTOM^+^ cells are present in the BSA sample at 14 DPI. **R)** Few tdTOM^+^ cells are localized to the P2 stump, whereas diffuse ACAN immunostaining caps the stump, indicative of fibrocartilage formation. Samples counterstained with DAPI. FGF8: n = 20 digits (5 mice) per time point; BSA: n = 20 digits (5 mice) per time point. Distal is to the right, dorsal is to the top. * = bead. Scale bars: A-C′, G, G’, K, K′, O, O′ = 200 μm; D–_F,_ H-J, L-N, P-*R* = 50 μm.

During development, articular cartilage and synovial cells express *Prg4* [49], and developmental lineage tracing studies demonstrate that the *Prg4*-lineage gives rise to cells that rearrange into each layer of the articular cartilage [75]. Given that *Prg4* is stimulated following FGF8 treatment (Fig. 3C and Fig. 6F and J), we questioned the spatial *Prg4* expression pattern and to what extent the induced-*Prg4*-lineage gives rise to structures in a regenerated joint. At 1 DPI, abundant clustered and punctate (arrowheads) *Prg4* transcripts were localized distal to and surrounding the P2 stump (*n* = 16 digits; 4 mice), whereas *Prg4* transcripts were absent in the BSA control (n = 16 digits; 4 mice) stump region (Fig. 7A, A’). By 5 DPI, *Prg4* expression was restricted to multiple cell layers surrounding the induced cavity (n = 16 digits; 4 mice) and absent in the BSA-treated stump (n = 16 digits; 4 mice) (Fig. 7B–B’). For lineage tracing, we utilized commercially available heterozygote *Prg4*^*GFPCreERt2*^ mice [31] crossed with *R26-tdTomato* mice, followed by P2 amputations at PN3 and FGF8 (*n* = 20 digits per time point; 5 mice per time point) or BSA (n = 20 digits per time point; 5 mice per time point) treatment at 4 DPA. At 1 DPI, mice were injected once with tamoxifen to label FGF8-induced *Prg4*^+^ cells, and tissues were collected at 5 and 14 DPI for histological and immunohistochemical analysis. At 5 DPI, histological analysis demonstrated that the cavity and cartilaginous nodule had formed following FGF8 treatment (Fig. 7C, D), whereas cavity and nodule formation was absent after BSA treatment (Fig. 7G, H). Immunostaining for the *Prg4*-lineage (tdTOM^+^ cells) in serial sections revealed robust tdTOM^+^ cells, encompassing the nodule and nascent EOC, the lining of the induced cavity, as well as the endogenous *Prg4* expression of the P1/P2 joint (open arrowhead) (Fig. 7C’, D′). We noted the central core of the nodule was not tdTOM^+^, and we speculate that at the time of tamoxifen treatment, those cells, or their progenitors, had already ceased *Prg4* expression (Fig. 7D, F). Few tdTOM^+^ cells were present at 5 DPI in the BSA treated stump, yet tdTOM^+^ cells were associated with the endogenous *Prg4* expression of the P1/P2 joint (open arrowhead) (Fig. 7G’,H′). Immunostaining for SOX9 and tdTOM at 5 DPI identified double-labeled individual cells (arrowheads) as well as a cluster of double-labeled cells within the nodule that surrounded the central core of SOX9^+^/tdTOM^−^ cells (Fig. 7E). Immunostaining for cell proliferation identified PCNA^+^/tdTOM^+^ cells scattered throughout the nodule but absent from the central core, and identified double-labeled cells in the nascent EOC (Fig. 7F, arrowheads). Conversely, Sox9 was localized only to the P2 stump growth plate after BSA treatment, with few tdTOM^+^ cells associated with the distal stump (Fig. 7I). BSA-treated digits showed broad PCNA expression both in the stump growth plate and the distal fibrotic cap, with nearly absent tdTOM immunostaining (Fig. 7J). By 14 DPI, FGF8-induced regenerates had undergone maturation and higher order patterning, including EOC chondrogenesis, expansion of the cavity, and ligament (arrowheads) and tendon (arrow) attachment to the nodule (Fig. 7K, L), whereas BSA-control digits were characterized by stump ossification and distal soft tissue fibrosis (Fig. 7O, P). Immunohistochemical analysis of a serial sectioned sample identified the *Prg4*-lineage had contributed to the regenerated nodule and cells associated with the ligament (arrowheads) and tendon (arrow) attachments to the nodule, as well as some cells within the EOC (Fig. 7K’, L’). Similar to 5 DPI, we noted the central core of the nodule was not tdTOM^+^ (Fig. 7L’). BSA-treated digits exhibited a paucity of tdTOM^+^ cells at 14 DPI (Fig. 7O’, P′). SOX9 and tdTOM staining revealed few doubled-labeled cells in the EOC (arrowheads), and several SOX9^+^/tdTOM^−^ cells (arrows) in the proximal portion of the EOC, but broad SOX9^+^/tdTOM^+^ cells scattered throughout the nodule (Fig. 7M). Conversely, Sox9 and tdTOM immunostaining were nearly absent after BSA treatment (Fig. 7Q). ACAN/tdTOM immunostaining revealed a similar staining pattern in the EOC; while distal EOC cells stained tdTOM^+^, few cells were ACAN^+^/tdTOM^+^ (arrowheads), and the proximal portion of the EOC stained ACAN^+^/tdTOM^−^ (Fig. 7N, arrows). Within the nodule, ACAN^+^/tdTOM^+^ cells were primarily localized to the periphery (Fig. 7N). After BSA treatment, tdTOM was largely absent and ACAN immunostaining was localized to the distal P2 stump, presumably associated with fibrocartilage formation as shown in Figs. 1H’ and 2I’ (Fig. 7R). Lastly, as a further control to test the effect of tamoxifen treatment and to assess if the reporter is leaky, corn oil treated *Prg4*^*GFPCreERt2*^;*R26-tdTomato* mice treated with FGF8 (*n* = 20 digits, 5 mice) were harvested at 21 DPI (Supplementary Fig. 7). Histological analysis and tdTOM immunostaining revealed no overt changes to the regenerative response and no tdTOM staining in the absence of tamoxifen. Taken together, these data support the conclusion that the FGF8-induced *Prg4*-lineage gives rise to many of the regenerated structures, including the nodule, cavity-lining cells, tendon and ligament cells that form an attachment to the nodule, as well as contribute, in part, to the EOC.

## 4. Discussion

There is a well-defined spectrum of regenerative competency within the mouse digit; distal P3 amputation results in robust bone and soft tissue regeneration, whereas progressively proximal P3 amputation initiates progressively reduced bone regeneration, and finally, amputations transecting P2 are non-regenerative [8,12,15,17,76,77]. In adult mouse digits, P2 amputation triggers a bone healing response reminiscent of the proximal bone fragment during fracture repair [17]. The amputated P2 bone transitions through the phases of inflammation, periosteal cartilaginous callus formation that forms a template for woven bone deposition, followed by secondary remodeling of the stump. The amputated ventral tendon undergoes a healing response that results in thin tendon fibers encasing the distal stump and inserting via an enthesis on the dorsal bone surface [17]. Conversely, neonate P2 amputations do not initiate periosteal callus formation, yet are more complicated due to the inclusion of the proximal growth plate during the wound healing response, thus development and regenerative failure occur simultaneously [16,22]. Taken together, P2 regenerative failure is not inert, but rather is a dynamic multi-tissue wound healing response that culminates in bone truncation and soft tissue fibrosis. Using the P2 amputation model, we have previously demonstrated robust composite tissue regenerative outcomes including BMP2-induced bone and tendon regeneration [16,20], and BMP9-induced synovial joint regeneration [22]. In the current study, we report that FGF8 is another potent inducer of synovial joint regeneration at P2 amputation wounds. FGF8 treatment functions to transition P2 fibrotic scarring into a multi-tissue regenerative response characterized by articular-like cartilage, synovial cavity, tendon, ligament, and bone regeneration.

Our results show that FGF8 induces a stepwise regenerative response by first stimulating synovial cavity and cartilaginous nodule formation, followed by the induction of an endochondral ossification center (EOC) that caps the distal stump and facilitates partial P2 bone regeneration. The nodule undergoes endochondral ossification centrally, whereas the cartilage adjacent to the synovial cavity matures into articular chondrocytes. Thus, FGF8 stimulates joint regeneration first and P2 bone regeneration secondarily. As such, the final regenerate is strikingly similar to that induced by the sequential treatment strategy of first treating the P2 wound with BMP2 to induce EOC formation, followed by BMP9 to induce synovial joint regeneration [22]. An intriguing conclusion here is that reversing the sequence of tissue morphogenesis, i.e., joint regeneration followed by stump regeneration versus stump regeneration followed by joint regeneration, does not have a discernable impact on regeneration and instead results in near identical regenerative outcomes. Importantly, after FGF8 treatment, *Bmp2* expression is unchanged at 24 h, yet is upregulated at 72 h post treatment. We suggest that the upregulation of *Bmp2* well after FGF8-induced joint morphogenesis is associated with the temporally staggered EOC formation and subsequent partial P2 bone regeneration. Taken together, FGF8 and BMP9 are each inducing regenerative programs to initiate joint regeneration with a similar phenotypic outcome. It is important to note that both the triangular P3 bone and the sesamoid bone are not restored in either of these regenerative responses, thus pointing to a higher-order regenerative program (i.e., instructive patterning cues) that continues to remain dormant, or is otherwise missing following FGF8 or BMP9 stimulation. An interesting conclusion here is that both regenerative programs may be relying on default positional information to pattern near-identical joint outcomes that is overall insufficient to pattern higher order structures, such as the triangular shaped P3 bone. Regardless, further studies are needed to compare and contrast the mechanisms that characterize both FGF8 and BMP9-induced joint regeneration. And given that multiple growth factors can induce joint regeneration, including FGF2 treatment [29], we also predict that other growth factors can induce joint regeneration at neonate P2 amputation wounds.

While it is noteworthy that a single growth factor can induce a composite tissue regeneration response, we initially surmised that FGF8 functioned to induce de novo interzone formation, which would then entirely coordinate joint regeneration. Yet, our data demonstrate that FGF8-induced joint regeneration utilizes few genes associated with joint and synovial cavity morphogenesis. Furthermore, this regenerative response is morphologically distinct from digit joint formation, as the phalangeal joints develop within interzones that essentially interrupt the cartilaginous anlagen [61], whereas this is not observed in FGF8-induced joint regeneration. The fact that induced joint regeneration does not recapitulate joint development, yet utilizes some genes, cells and structures (i.e., chondrocytes and the synovial cavity) of development, should not be surprising within the greater context of mammalian digit regeneration. For example, P3 regeneration utilizes osteoblasts to restore the bone, yet does not recapitulate the endochondral ossification of P3 development, as P3 regeneration is carried out via intramembranous ossification [8] that involves a genetic program distinct from development [62].

During joint development and maturation, interzone cells and surrounding proximal and distal cell populations contribute to the overall higher order structure of the joint, including the synovial cavity, articular cartilage, ligaments, and the joint capsule [58,78–80]. P2 amputation removes the articular cartilage, the joint cavity, and severs the tendon and ligament, yet seemingly leaves the surrounding proximal cell populations intact. FGF8 (as well as BMP9) may be targeting the remaining proximal cell populations to organize them into a joint. Indeed, our previous BMP9-induced joint regeneration studies have shown a greater efficiency of joint regeneration in the neonate digits than in adult digits (62 % compared to 14 %), and thus points to either the enhanced cellular plasticity in younger animals [81], and/or the necessity for remaining developmental cell populations to induce joint regeneration at a greater frequency. The current studies focused on the neonate digit response to FGF8 treatment, yet we expect a similar decline of FGF8-induced joint regeneration efficiency as the animal matures. While more studies are required to tease apart regenerative capacity across the lifespan, including future studies on FGF8-induced joint regeneration in mature mice, the fact that a joint can be induced in a mature animal using BMP9 [22] is a proof-of-concept that joint regeneration can indeed occur in the absence of concurrent development. Nonetheless, developing an understanding of joint morphogenesis is an invaluable tool for predicting the cell populations that participate in joint regeneration. For example, developmental studies have demonstrated that the *Prg4*-lineage gives rise to individual chondrocytes whose daughter cells orient themselves into a stack to contribute to all layers of the articular cartilage [75]. Using a similar approach in which we labeled the FGF8-induced *Prg4*-lineage, our findings demonstrate that the stump *Prg4*-lineage gives rise to a handful of regenerated structures, including the nodule chondrocytes and attached tendon and ligament cells, the cavity lining cells, as well as limited contribution to the EOC.

Collectively, our findings support the conclusion that cells at otherwise non-regenerative amputation wounds possess latent capacity for organized composite tissue regeneration, including bone, joint, tendon and ligament regeneration. Under normal amputation conditions, this regenerative capacity is not tapped into yet can be stimulated with appropriate growth factor treatment. Nevertheless, it is important to note there are several limitations in this study. First, these studies did not focus on the release rate or dose of FGF8 supplied by the microcarrier bead. As such, the observed joint ratio of ~61.5 % could potentially be enhanced with a superior growth factor vehicle. In line with this, what drives the observed joint ratio of ~61.5 % is unknown. We speculate that several scenarios could be occurring; 1) suboptimal bead placement or movement of the bead following implantation, 2) dislodging of the bead prior to joint initiation, or 3) given that the P2 amputation wound is heterogenous, the appropriate dose of FGF8 may not target the appropriate cells to induce joint regeneration. Understanding this ratio is the subject of future studies. Second, given that scRNA-seq was carried out only once, it is possible that sampling artefacts could account for enrichment of Fibroblast cluster 1.

## Supplementary Material

Supplementary Table 2

Supplementary Table 1

ARRIVE Guidelines

Supplementary Material

Supplementary data to this article can be found online at https://doi.org/10.1016/j.bone.2025.117663.

## Data Availability

The datasets analyzed during the current study are available from the corresponding author on reasonable request. Single cell RNA-Seq data were deposited into the Gene Expression Omnibus database under accession number GSE294048 and are available at: https://www.ncbi.nlm.nih.gov/geo/query/acc.cgi?acc=GSE294048
