# Supplementary material for: FGF8 induces bone and joint regeneration at digit amputation wounds in neonate mice
Source: Bone. Author manuscript; Available in PMC 2026 Mar 27. (PMC13029017; doi:10.1016/j.bone.2025.117663)
Supplement: Supplementary Material [file NIHMS2155141-supplement-Supplementary_Material.docx]

**
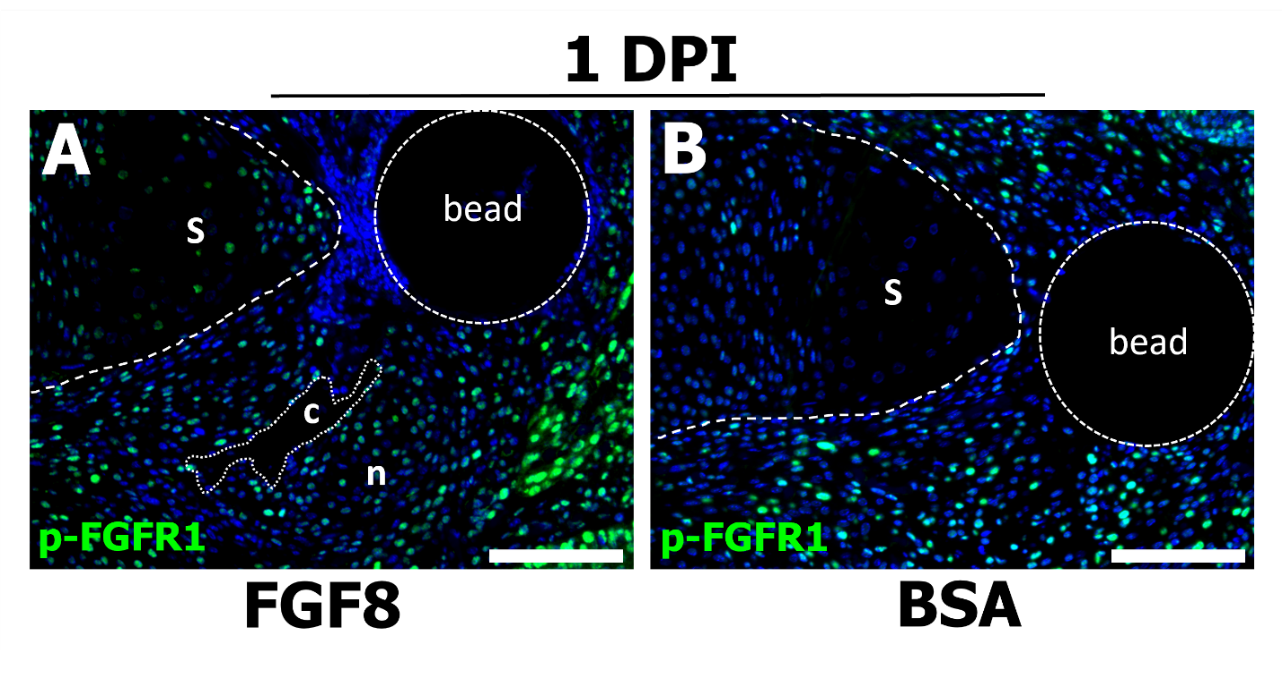
Supplementary Figure 1.** Immunostaining for phospho-FGFR1 at 1 day post FGF8 **(A)** and BSA **(B)** treatment. S = stump, c = cavity, n = nodule. Distal is to the right, dorsal is to the top. Scale bar = 50 µm.


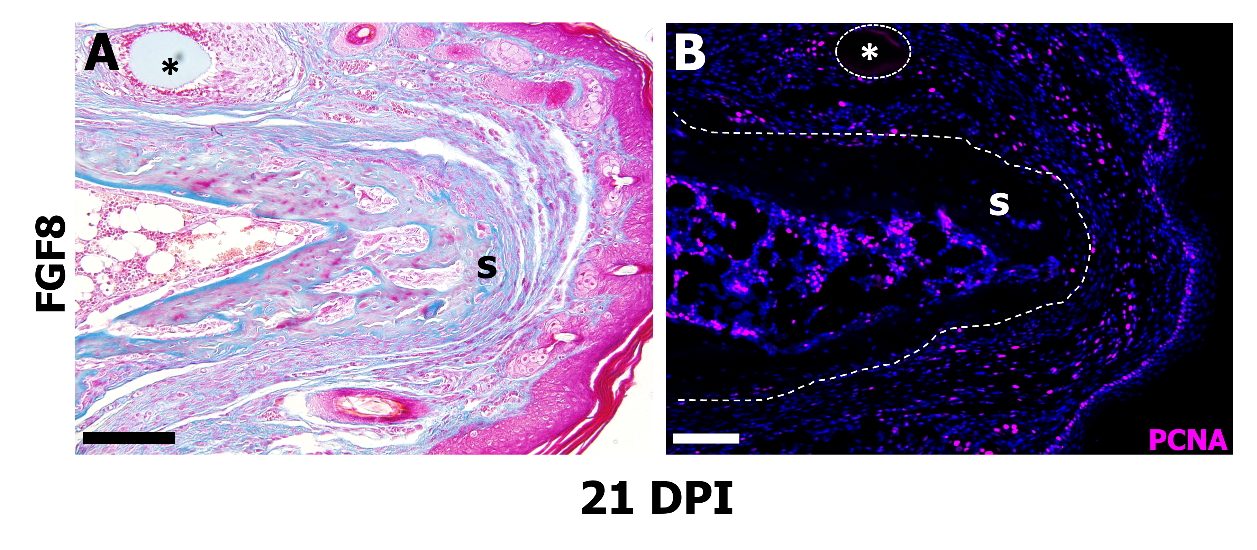


**Supplementary Figure 2.** **A)** Mallory trichrome staining of non-regenerative FGF8 treated digit at 21DPI. **B)** Immunostaining for PCNA at 21 DPI. S = stump, * = bead. Distal is to the right, dorsal is to the top. Scale bars = 200 µm.


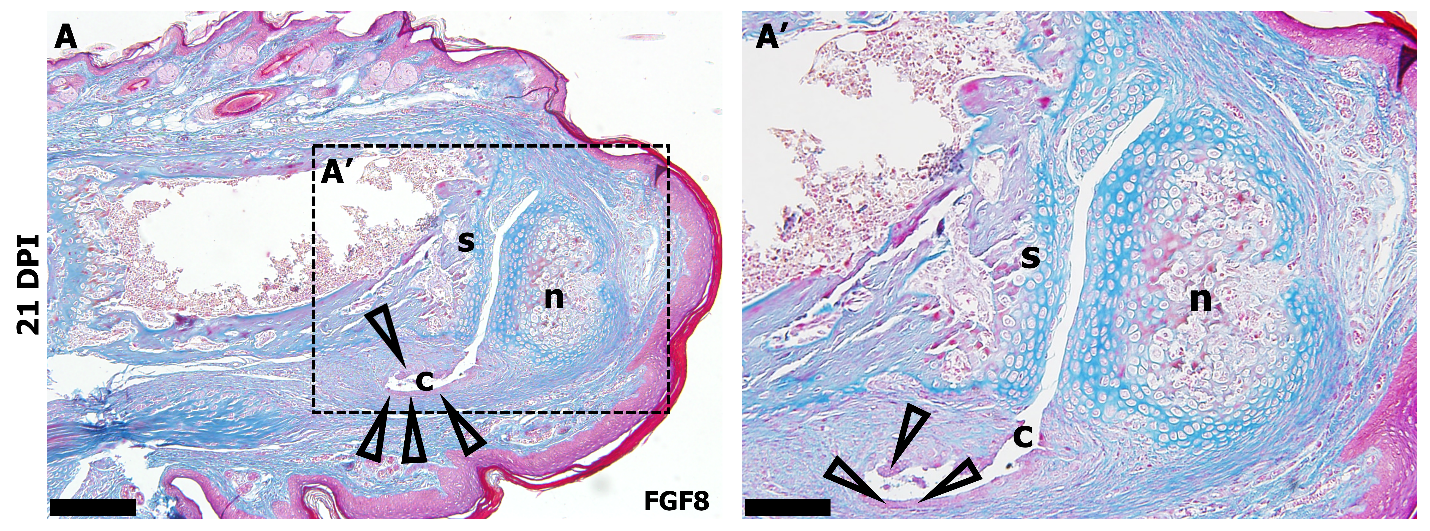


**Supplementary Figure 3. A-A’)** Mallory trichrome staining of a FGF8 treated digit **A)** and inset **A’)** at 21DPI. S = stump, c = cavity, n = nodule. Open arrowheads indicate cavity lining Prg4+ cells shown in Fig. 1I. Distal is to the right, dorsal is to the top. Scale bars A = 200 µm; A’ = 100 µm.


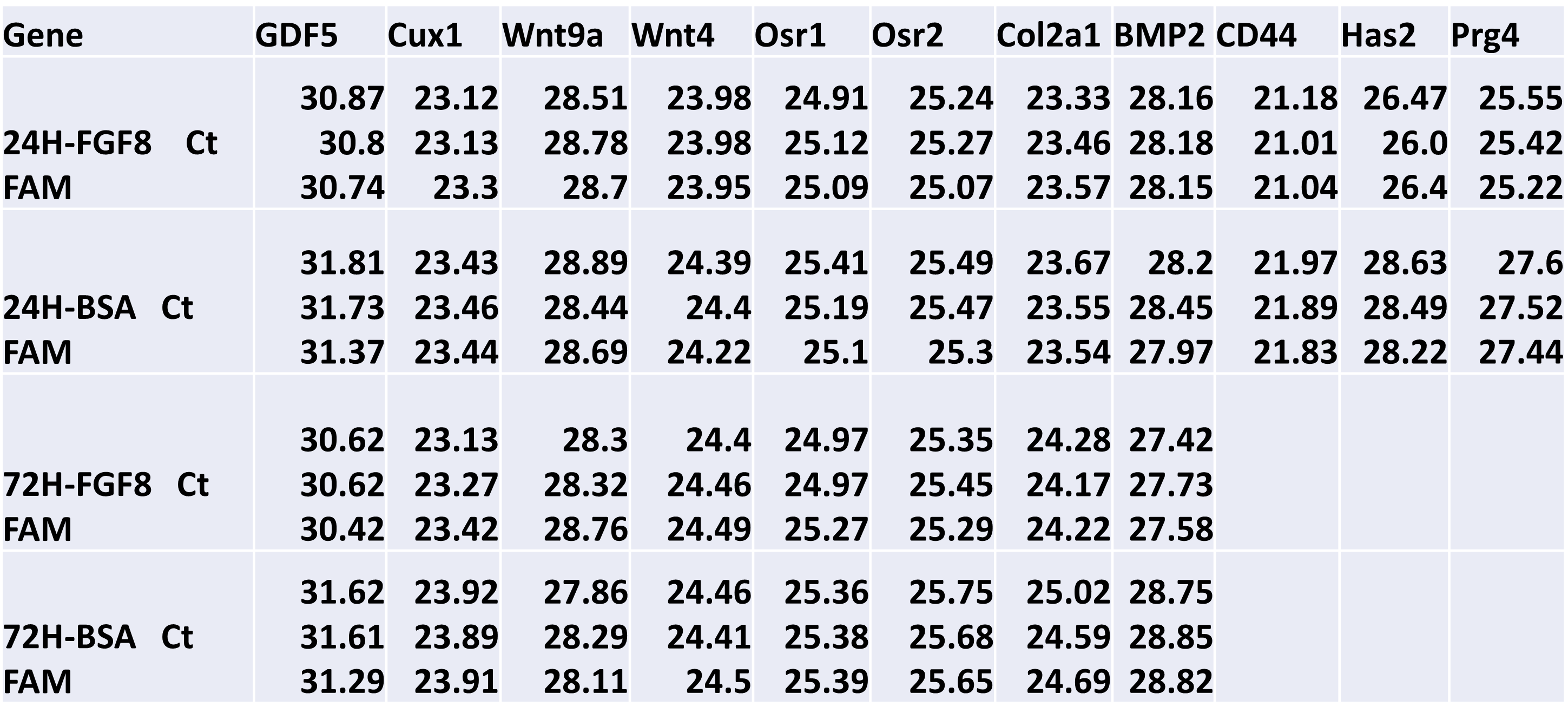


**Supplementary Figure 4.** Cycle threshold (Ct) values of genes assayed by qRT-PCR.


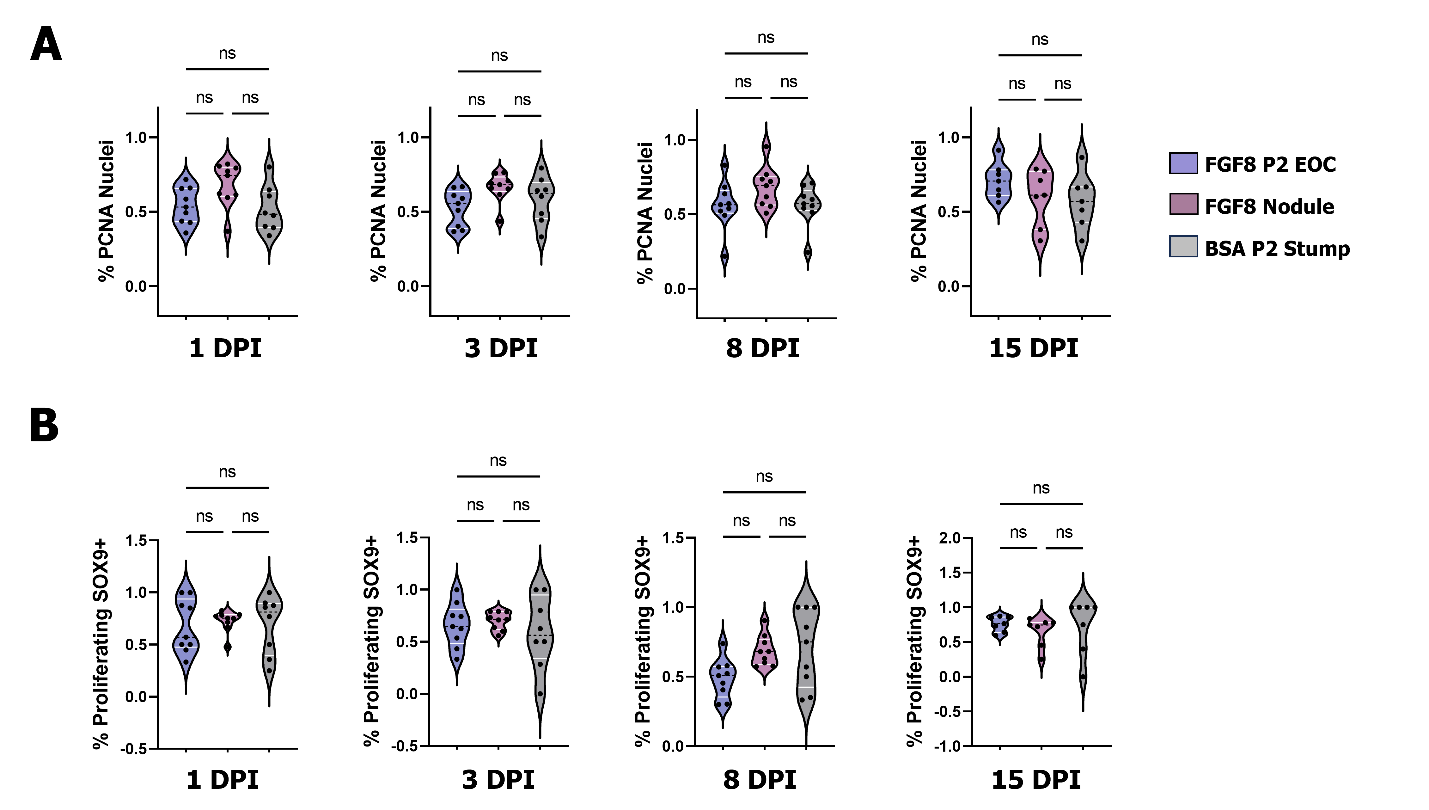


**Supplementary Figure 5. A)** Quantification of cell proliferation and **B)** proliferating Sox9+ cells at 1, 3, 8, and 15 DPI. 1 DPI: FGF8 = 9 digits (4 mice); BSA = 8 digits (4 mice). 3 DPI: FGF8 = 9 digits (4 mice); BSA = 8 digits (4 mice). 8 DPI: FGF8 = 9 digits (5 mice); BSA = 9 digits (4 mice). 15 DPI: FGF8 = 7 digits (4 mice); BSA = 7 digits (4 mice). One-way ANOVA; ^ns^*p* > 0.05.


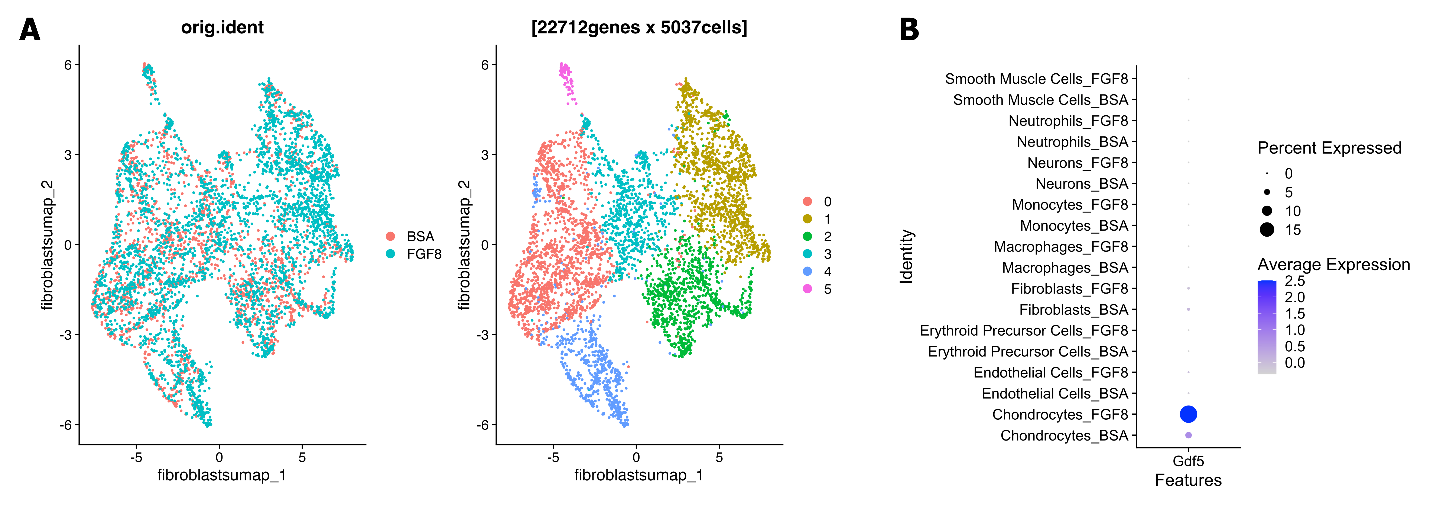


**Supplementary Figure 6. A)** Umap plots of digit fibroblasts and fibroblast subclusters at 24 h post FGF8 or BSA treatment. **B)** Gdf5 expression in all cell types, shown as dot plots.


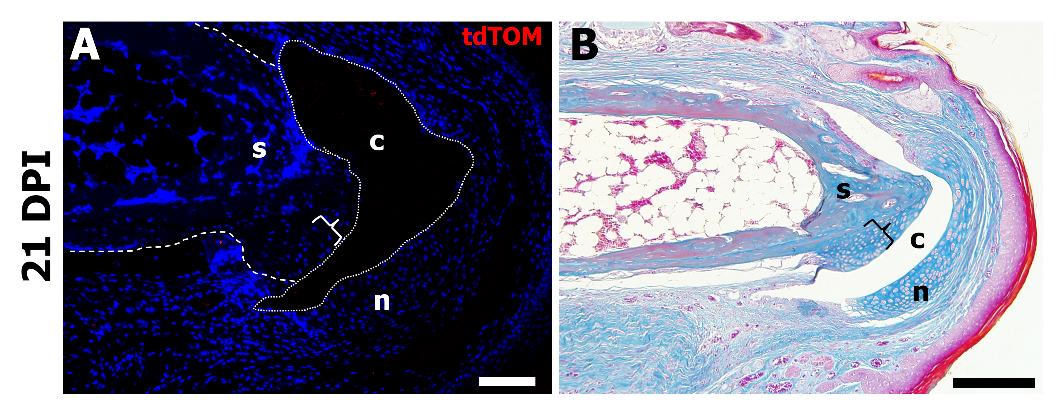


**Supplementary Figure 7.** **A)** Immunostaining for tdTOM at 21DPI in FGF8-treated corn oil control lineage tracing digit. **B)** Mallory staining of FGF8 treated corn oil control lineage tracing digit at 21 DPI. S = stump, c = cavity, n = nodule, bracket = endochondral ossification center. Distal is to the right, dorsal is to the top. Scale bars = 200 µm.
